# Advances in Pancreatic Cancer Treatment by Nano-Based Drug Delivery Systems

**DOI:** 10.3390/pharmaceutics15092363

**Published:** 2023-09-21

**Authors:** Cláudia Viegas, Ana B. Patrício, João Prata, Leonor Fonseca, Ana S. Macedo, Sofia O. D. Duarte, Pedro Fonte

**Affiliations:** 1 Faculty of Medicine and Biomedical Sciences (FMCB), University of Algarve, Gambelas Campus, 8005-139 Faro, Portugal; viegas.claudiasofia@gmail.com; 2 Center for Marine Sciences (CCMar), University of Algarve, Gambelas Campus, 8005-139 Faro, Portugal; 3 iBB—Institute for Bioengineering and Biosciences, Instituto Superior Técnico, Universidade de Lisboa, Av. Rovisco Pais 1, 1049-001 Lisboa, Portugal; ana.patricio@tecnico.ulisboa.pt (A.B.P.); sofia.duarte@tecnico.ulisboa.pt (S.O.D.D.); 4 Associate Laboratory i4HB—Institute for Health and Bioeconomy at Instituto Superior Técnico, Universidade de Lisboa, Av. Rovisco Pais, 1049-001 Lisboa, Portugal; 5 LAQV, REQUIMTE, Applied Chemistry Lab—Department of Chemical Sciences, Faculty of Pharmacy, University of Porto, Rua de Jorge Viterbo Ferreira 228, 4050-313 Porto, Portugal; 6 Department of Chemistry and Pharmacy, Faculty of Sciences and Technology, University of Algarve, Gambelas Campus, 8005-139 Faro, Portugal

**Keywords:** pancreatic cancer, polymer nanoparticle, lipid nanoparticle, hybrid nanoparticle, inorganic nanoparticle, nanocarrier

## Abstract

Pancreatic cancer represents one of the most lethal cancer types worldwide, with a 5-year survival rate of less than 5%. Due to the inability to diagnose it promptly and the lack of efficacy of existing treatments, research and development of innovative therapies and new diagnostics are crucial to increase the survival rate and decrease mortality. Nanomedicine has been gaining importance as an innovative approach for drug delivery and diagnosis, opening new horizons through the implementation of smart nanocarrier systems, which can deliver drugs to the specific tissue or organ at an optimal concentration, enhancing treatment efficacy and reducing systemic toxicity. Varied materials such as lipids, polymers, and inorganic materials have been used to obtain nanoparticles and develop innovative drug delivery systems for pancreatic cancer treatment. In this review, it is discussed the main scientific advances in pancreatic cancer treatment by nano-based drug delivery systems. The advantages and disadvantages of such delivery systems in pancreatic cancer treatment are also addressed. More importantly, the different types of nanocarriers and therapeutic strategies developed so far are scrutinized.

## 1. Introduction

Pancreatic cancer is among the most prevalent forms of cancer and is a leading cause of death, affecting more men than women. Its 5-year survival rate is less than 5%. According to GLOBOCAN data from 2020, this type of cancer accounted for over 466,003 deaths annually, making up 4.7% of all deaths. It ranks as the 7th leading cause of cancer-related deaths for both genders [1]. The 5-year survival rate for pancreatic cancer is estimated to be less than 5%. The incidence and mortality rates are observed to increase with age and are more prevalent in males compared to females. In 2023, it is projected that the number of fatalities in the United States due to this cancer will rise to 50,550, according to Cancer Statistics [1,2,3]. This is due to the lethal nature of this type of cancer since it can rapidly spread to the lymphatic system and distant organs, being mostly asymptomatic up to stage IV. The presence of imperceptible metastases when the diagnosis occurs, combined with the inefficiency of the treatments that currently exist due to the limited drug delivery, deficient drug penetration in an abundant pancreatic stroma tumor, and the intrinsic resistance of the tumor cells to the chemotherapeutic agents, contribute to the high rate of mortality [3,4].

In recent decades, pancreatic cancer-related mortality has also increased in both developed and developing countries. Its incidence varies across regions, with Asia and Europe reporting the highest rates (47.1% and 28.3% of cases, respectively) in 2020. Limited access to healthcare in developing countries contributes to these variations [5,6,7,8,9].

Conventional treatment protocols for pancreatic cancer are based on the specific cancer type and disease stage. Typically, they involve a combination of local surgery along with other therapeutic strategies, such as chemotherapy and radiotherapy. So, chemotherapy is currently the most common treatment option, although it allows for minimal survival. Gemcitabine (GEM) is the first-line chemotherapy agent, prolonging overall survival in only 6 to 12 weeks. However, its benefits are compromised by its low half-life and low relative concentration around the tumor tissue. Moreover, a significant proportion of pancreatic cancer patients are not eligible for surgical intervention due to delayed diagnosis, early metastasis, and extensive local tissue invasion. Also, the absence of biomarkers, high recurrence rates, and chemotherapeutic resistance are significant contributors to the elevated mortality rates observed in pancreatic cancer patients. So, these strategies demonstrate limited efficacy in enhancing patient survival rates. Consequently, new and effective therapies are urgently needed [10,11,12,13,14,15].

Numerous efforts have been focused on targeting tumor stroma and vasculature to enhance the delivery and effectiveness of therapeutic agents for pancreatic cancer treatment. Furthermore, the highly desmoplastic and immunosuppressive tumor microenvironment of most pancreatic tumors contributes to their poor responsiveness to therapeutic agents. To address these challenges that hinder drug delivery and efficacy, significant advancements have been made in the field of nanotechnology over the past few decades. These advancements introduce robust methods for efficient drug delivery to pancreatic tumors, overcoming the aforementioned limitations [13,15,16].

Nanotechnology is a multidisciplinary science that addresses the development and preparation of systems on a nanometer scale. This science involves the utilization of particles ranging in size from 1–1000 nm. Depending on their size, these particles can exhibit varied properties in terms of stability, reactivity, and their capacity to interact with other molecules and biological systems [17,18]. Therefore, this type of technology gives the possibility to develop new methods that can be applied positively in various scientific areas. The integration of nanotechnology into medicine has emerged as a groundbreaking interdisciplinary research area. Over the past decade, its prominence has grown significantly due to its added value in both the diagnosis and treatment of various pathologies [19,20].

Many research studies have been conducted involving nanotechnology and pancreatic cancer. Various types of nanoparticles, including lipid, polymeric, and metallic nanoparticles, have been developed to enhance the treatment efficacy for pancreatic tumors [21]. However, toxicity remains a primary concern with these formulations, along with challenges related to their long-term stability [22,23].

Therefore, the main aim of this review is to make an overview of the nanocarriers developed so far for pancreatic cancer treatment. Their advantages and disadvantages are debriefed. Also, some toxicity concerns about such systems and the products with the potential to improve patients’ quality of life are fully disclosed.

## 2. Methodology

To address the mentioned concern, a comprehensive search was conducted in the PubMed and Web of Science databases for articles published within the last decade. Keywords related to pancreatic cancer, nanotechnology, nanomedicine, nanoparticles, nano-based, polymer, lipid, hybrid, inorganic, therapy, and treatment were employed in various combinations. Additionally, citations from the selected articles were included as supplementary sources. Just articles published in English with available full text were considered, without restrictions on article types (original articles, reviews, etc.), subjects involved in the research (e.g., humans, mice), experimental conditions (in vitro or in vivo), and population size.

## 3. Anatomophisiology of the Pancreas

The pancreas is an acinar tubular gland located in the retroperitoneal space, between the large curve of the stomach and the duodenum, arranged transversely in the upper wall of the abdomen. It has an elongated and flat structure, which measures approximately 15 cm in length and weighs 85–100 g [24,25].

**Figure 1:**
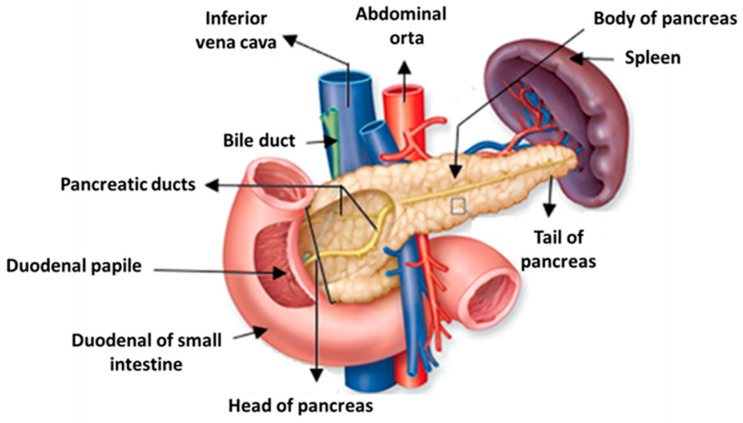
Anatomy of the pancreas. Adapted with permission from [27].

This organ consists of the head, which is in contact with the duodenal loop and separated from the body of the pancreas through an isthmus, a restricted zone bounded by two cracks of the body, a slightly oblique part from below to and from right to left, disposed frontally to the aorta and the inferior vena cava, and of the tail, which is in contact with the spleen and is lined by the parietal peritoneum [26], as shown in Figure 1 [27].

The pancreas is a gland with two functions: endocrine and exocrine. The exocrine pancreas secretes gastric enzymes into the digestive tract, which help in the food breakdown process. This fluid is a mixture of hydrochloric acid, gastric juice, and digestive enzymes such as trypsin, amylase, and lipase. Its role is to surround the partially processed food, known as the food bolus, and convert it into a substance called chyme. It reaches the duodenum through the Wirsung duct, which leads to the Vater ampule, and the Santorini duct, which flows 3 to 4 cm above. Pancreatic acini releases pancreatic juice to complete the process of chyme digestion in the duodenum. The main components of pancreatic juice are water, salts, bicarbonate, and various digestive enzymes. These components, namely the bicarbonate ions, are responsible for neutralizing the acid in the chyme and thus protecting the intestinal wall. In addition, this change creates a favorable environment for pancreatic enzymes to function properly [28].

The enzymes present in the pancreatic juice are:

- Proteolytic enzymes, whose function is the digestion of proteins. They are divided into exopeptidases (such as carboxypeptidase), which act on the chemical bonds between amino acids, from a terminal end of the protein, and into endopeptidases (such as chymotrypsin and trypsin), which degrade proteins by cleaving chemical bonds between the amino acids of the protein molecule [25,29];
- Glycolytic enzymes, or amylases, contribute to the digestion of carbohydrates and sugars. These enzymes hydrolyze the α-bonds in the starch chain, converting them into simple sugars (glucose and maltose). These sugars can then easily cross the intestinal mucosa and enter the bloodstream [25,30];
- Lipolytic enzymes, or lipases, whose function is to digest lipids or fats. The lipases hydrolyze the fats, transforming them into glycerol-free fatty acids, easily assimilated by cells. In addition to this function, they also break down neutral fats or triglycerides into fatty acids and glycerin [25,26,27,28,29,30,31].
- Nucleases enzyme, that promote the digestion of nucleic acids. Ribonuclease cleaves RNA molecules in the sugar ribose and the nitrogenous bases adenine, cytosine, guanine, and uracil, while the deoxyribonuclease digests the DNA molecules in the sugar deoxyribose and the nitrogen bases cytosine, adenine, guanine, and thymine. There are enzymes of two types (α and β) that catalyze the hydrolysis of the phosphodiester bonds [25,32].

The endocrine pancreas plays a pivotal role in maintaining blood glucose balance. It is essential to regulate glucose levels in the bloodstream to ensure a consistent and steady supply of glucose to cells. Elevated levels of glucose can cause damage to the kidneys, eyes, and other tissues. So, the pancreas secretes two antagonistic hormones that control this homeostasis: glucagon and insulin [33]. Glucagon is produced by α-cells and accumulates within the secretory granules from which it is released by exocytosis at the time of hypoglycemia. In the liver, hepatocytes recognize this through a specific receptor. This recognition triggers a series of phosphorylation reactions that activate the enzymes responsible for glycogen breakdown and glucose synthesis, leading to an increased release of glucose into the bloodstream. Glucagon hormone also stimulates the fat tissue to transform triglycerides into glucose. Finally, this hormone promotes amino acid uptake and active phagocytosis mechanism, along with others [34].

In contrast to glucagon, insulin functions to reduce blood glucose levels after food consumption by facilitating the uptake of glucose by the liver, muscles, and adipose tissues. Insulin is produced in β-cells, and when there is a peak of glucose in the blood, insulin is released to the systemic circulation, increasing the glucose uptake by cells by binding to the membrane receptor tyrosine kinase. This receptor activates the exocytosis of the GLUT4 transporter molecule’s specific glucose tolerance. When the extracellular concentration of glucose is reduced, GLUT2 is transported back into the cell by endocytosis and stored in vesicles for later use [32,35].

Insulin also stimulates the intracellular use of glucose and facilitates its transformation into glycogen (in the liver or muscles) or into triglycerides (adipose tissue) in a process named glycolysis. In addition, insulin stimulates lipid metabolism, favoring the passage of free fatty acids from plasma to adipocytes, which convert them into triglycerides, reducing the mobilization of fats and inhibiting their oxidative dissolution. It also acts on the metabolism of proteins, facilitating the transport of amino acids to cells [25,35,36].

Insulin secretion is mediated by glycemia through a negative feedback mechanism. β cells are overly sensitive to blood glucose levels, so by massively flowing through the membrane, it causes a series of biochemical reactions that end with the depolarization of cells. By activating a system of microtubules and microfilaments, the flow of Ca^2+^ ions promotes the excretion of this hormone [25,37].

The pancreatic functions are controlled by the autonomic nervous system (ANS) and the endocrine hormone system. The ANS is constituted by sympathetic and parasympathetic innervation. The sympathetic innervation acts in situations of stress, fear, emergency, or excitation, while the parasympathetic innervation acts in the opposite situations, namely during rest and digestion. The sympathetic innervation acts by stimulating the α cells of the pancreas to secrete glucagon into the bloodstream, which in turn stimulates the liver to initialize glycogen cleavage into small glucose molecules. Subsequently, glucose is released into the bloodstream and reaches the cardiac and skeletal muscles. The pancreas β cells are also activated by the sympathetic innervation to reduce the secretion of glucose and insulin, which counteracts the effect. The parasympathetic innervation, opposing the sympathetic innervation, stimulates the release of insulin and pancreatic secretions [38].

Secretin and cholecystokinin (CCK) are the two hormones secreted by the endocrine system to regulate digestive function. Secretin and CCK are produced by the cells of the duodenum lining. The former is produced to respond to the arrival of the chyme, and it induces the secretion of bicarbonate and water by the pancreas while it also inhibits the formation of gastrin by the stomach. Gastrin is produced in response to the presence of proteins and fats in the chyme. Once released, it circulates through the bloodstream and binds to pancreatic acini. This binding stimulates these cells to produce and release pancreatic secretions rich in digestive enzymes. These enzymes then help break down protein molecules into peptides and convert lipid molecules into soluble microdroplets, facilitating their absorption by intestinal cells [39,40].

## 4. Pancreatic Cancer

Pancreatic cancer ranks among the principal causes of cancer-related mortality, characterized by a reduced 5-year survival rate. The disease starts without any precise early signs or symptoms, and its expression will vary depending on the position of the tumor inside the organ. Around 50% of patients have icterus, which is more frequently observed when the tumor is in the head of the pancreas as a result of the blocking of the adjacent biliary system [41].

In addition, pancreatic cancer may manifest with other symptoms, including abdominal discomfort, nausea, and weight loss. When tumors spread to other organs, duodenal obstruction, gastrointestinal bleeding, and pancreatic duct obstruction can lead to steatorrhea. Early manifestations of the disease have been associated with hyperglycemia and diabetes mellitus. Advanced pancreatic cancer may present with ascites, pain, impaired liver function, hyperglycemia, anemia, and depression. These various symptoms further contribute to the complexity of diagnosing and managing pancreatic cancer [42,43].

Certain risk factors for pancreatic cancer, such as smoking habits, can be modified, while others remain unclear scientifically. Factors like diets rich in red and processed meats and low in fruits and vegetables, physical inactivity, and coffee consumption have been associated with pancreatic cancer. Recent studies have demonstrated an association between alcohol overuse and pancreatic cancer. This association is probably mediated through alcohol involvement in the development of chronic pancreatitis and cirrhosis, which are established risk factors for this particular cancer type. However, factors like age, family history, and type II diabetes are non-modifiable risk factors. Pancreatic cancer is more prevalent in individuals over 45 years of age, with males having a slightly higher risk than females. Additionally, African Americans are more vulnerable to pancreatic cancer than Caucasians. However, the reason behind the higher occurrence of type II diabetic individuals is still unknown. So, understanding and managing these risk factors can contribute to better preventive strategies for pancreatic cancer [41]. However, the presence of one or several risk factors characteristic of a pathology does not ensure the development of the disease [44]. Approximately 5% to 10% of pancreatic cancer patients have a family history of the disease. People having the *BRCA2* mutation, known for increased risk of breast and ovarian cancer, are now identified as having higher susceptibility to pancreatic adenocarcinoma. Besides *BRCA2*, other genes with variants associated with elevated pancreatic cancer risk include *BRCA1*, *MLH1*, *MSH2*, *PRSS1* (linked to familial pancreatitis), *STK11*, *PALB2*, *ATM*, *CDKN2A*, *APC*, *MSH6*, and *PMS2* [25,42,43].

**Figure 2:**
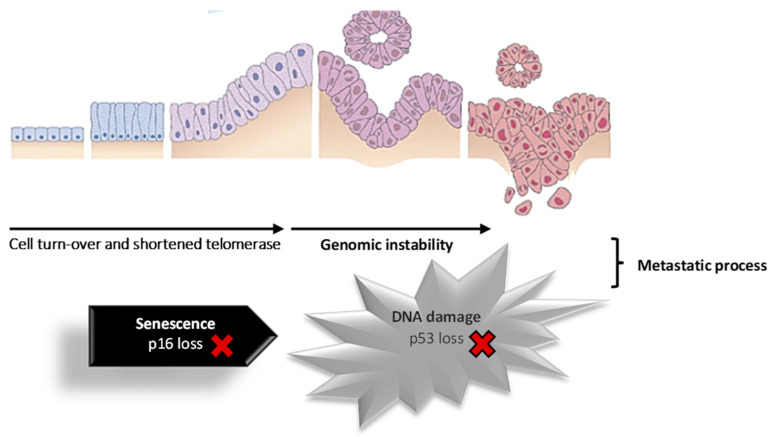
Schematic representation of the cell progression to pancreatic cancer. Adapted with permission from [49].

About 80% of cases of pancreatic cancer develop in the exocrine portion of the pancreas, and around 75% of cancers in the exocrine pancreas are situated within the head, 15 to 20% in the body, and only 5 to 10% in the tail [2,45]. Pancreatic cancer situated in the exocrine portion is predominantly diagnosed as ductal adenocarcinoma, accounting for approximately 95% of cases [46,47]. These primarily originate from the pancreatic ducts and, less commonly, from acinar cells (acinar cell carcinoma). Acinar cell carcinoma is distinguished by prominent acinar cell differentiation, cytoplasmic granules, and a prominent single nucleus [48]. Figure 2 shows the progression of normal pancreatic duct epithelium to pancreatic adenocarcinoma through early events (shortened telomerase, *KRAS* mutation, *p16* loss) and at a more advanced stage (*p53* loss, *SMAD4/DPC* gene loss). Pancreatic cancer is the result of hereditary mutations in cancer-related genes, including oncogenes, tumor suppressor genes, cell cycle genes, genes involved in apoptosis, and genome maintenance genes. Additionally, cell turnover, telomerase shortening, and genomic instability can contribute to the transformation of pancreatic epithelial cells into tumor cells [25,49].

Pancreatic intraepithelial neoplasm (PanIN) is usually located in the small pancreatic duct and is divided into 3 groups by epithelial atypia, PanIN-1 (minimum atypia), which also has 2 subgroups (i.e., PanIN-1A (flat type) and PanIN-1B (papillary type)), PanIN-2 and PanIN-3 (limited atypia). This type of neoplasm is associated with invasive carcinoma and chronic pancreatitis, and HER-2/neu expression is 82% in PanIN 1A, 86% in PanIN 1B, and 92% in PanIN-2 [50]. Pancreatic cysts are common, some of which are curable precursors of a possible ductal adenocarcinoma. Within these neoplasms, we first have the intraductal papillary mucinous, located in the exocrine pancreas. This type of lesion is defined by non-invasive productions of papillary mucin and arises in the larger pancreatic ducts. Because of their large size, they easily have been detected in imaging tests, and the evolution to more invasive cancer can be prevented [25,46,47,51,52].

Secondly, mucosal cystic neoplasms (MCNs) are well-defined tumors with mucin-producing cysts and septation with distinctive ovarian-like stroma without communication with the ductal system. It is usually a single lesion consisting of a thick fibrous wall. This type of neoplasm can be invasive and non-invasive. Non-invasive MCNs are subdivided into MCNs with low-grade dysplasia, moderate dysplasia, or high-grade dysplasia [47,53]. Thirdly, there is the serous cystadenoma SCAs, in which the cysts are mostly benign and slow-growing, coated by non-mucinous epithelium. Finally, solid pseudopapillary neoplasia is exceedingly rare. This type of lesion usually has a favorable prognosis, although it is mostly malignant [25,46,54,55,56].

Endocrine or neuroendocrine tumors (NETs) are relatively rare, accounting for less than 5% of all pancreatic cancers. These tumors can be either benign or malignant, and at the microscopic level, their appearance may be similar, making them challenging to identify. Malignancy is typically diagnosed when it metastasizes to other organs. Diverse types of NETs exist:

- Functional NETs: Approximately 50% of neuroendocrine tumors produce hormones that are released into the bloodstream, leading to the onset of symptoms (for example, gastrinomas, insulinomas, glucagonomas, somatostatinomas, VIPomas—vasoactive intestinal peptides, and the PPomas—pancreatic polypeptides).
- Non-functional NETs: This type of tumor typically does not produce hormones in levels high enough to cause noticeable symptoms, which makes them more likely to develop into cancer as they remain asymptomatic for a longer period.
- Carcinoid tumors: This type of tumor does not often originate in the pancreas, as they are more commonly found in other parts of the digestive system. These tumors typically produce serotonin (5-HT) or its precursor, 5-hydroxytryptophan (5-HTP).
- Pancreatoblastomas: This is an uncommon solid cell neoplasm, and their exact locations are not yet well characterized. These tumors comprise multiple cellular components, including the acinar component [25,57,58,59].

The American Joint Committee on Cancer has developed a tumor-nodule-metastasis classification system for the assessment of pancreatic cancer stage and type. The evaluation parameters include tumor size and its association with blood vessel involvement, leading to tumor characterization as TX to T4. The extent of lymph node involvement determines nodal classification from NX to N1. The presence or absence of identifiable metastases in distant organs defines the metastatic category as M0 or M1, respectively (Table 1 and Table 2) [41].

Improvements in the expertise of the pathogenesis of pancreatic cancer have been increasing in the last decade, thus contributing to the development of more effective diagnostic methods and therapies. Numerous subsets of genes have been identified to undergo activation or deactivation during pancreatic cancer progression. Activation of oncogenes (point mutation and amplification) and inactivation of tumor suppressor genes initiate the development of pancreatic cancer. Despite the complexity of all the genetic changes mentioned earlier, they collectively constitute a fundamental set of processes crucial for comprehending pancreatic cancer [47,60,61]. Pancreatic cancer is caused by somatic mutations, genetic alternations, and the germ line. There are sixteen identified mutated oncogenes, including *KRAS*, *TP53*, *CDKNA2A*, *SMAD4*, *MLL3*, *TGFBR2*, *ARID1A*, *SF3B1*, *EPC1*, *ARID2*, *ATM*, *ZIM2*, *MAP2K4*, *NALCN*, *SLC16A4*, *MAGEA6* [46,49,54].

The *KRAS* oncogene frequently undergoes mutation, mainly at codon 12 and occasionally at codons 61 and 13, leading to alterations in 90% of pancreatic cancer cells, with 20% of these affecting the entire body. This mutated oncogene exerts negative effects on cell survival and functions, including cell differentiation and proliferation [55]. A mutation in KRAS induces the development of ductal precancerous formations and triggers hyperplastic multifocal focus in the pancreatic duct. Additionally, it can activate multiple signaling pathways, such as the P13K-AKT pathway, which impacts cell survival and mobility; the MEK and ERK1/2 pathway, affecting angiogenesis, cell proliferation, cellular apoptosis, cancer cell migration, and cell cycle regulation; the notch pathway, influencing cell proliferation, cell differentiation, and cellular apoptosis; and the Hedgehog pathway, contributing to metastasis. Furthermore, the activation of *STAT3* has been identified in pancreatic cancer patients, and inhibitors targeting this oncogene are already being utilized in the treatment of this cancer [25,62].

MiRNAs are also implicated in the progression of pancreatic tumors and have incredibly important oncogenic functions. Between the 1000 existent miRNAs, pancreatic cancer functions are regulated by miRNA-196a, miRNA-190, miRNA-186, miRNA-200b, miRNA-15b, miRNA-95, miRNA-21, miRNA-155, miRNA -221 and miRNA-222 [63].

Tumor suppressor genes have the function of protecting the cell cycle or promoting apoptosis of tumor cells. The TP53, also known as the p53 protein, is encoded by a tumor suppressor gene and performs a key role in G2-M phase progression, regulating the G1-S checkpoint of the cell cycle. The expression of the *TP53* gene and the action of its protein are activated by cellular stress signals: nutritional and oxidative stress from reactive oxygen species (ROS), hypoxia, activation of oncogenes, and DNA damage. Thus, it induces apoptosis, regulates senescence, repairs DNA, and alters cellular metabolism if errors occur. A mutation in its gene by missense conjugation mutations with loss of the remaining allele results in the inactivation of this protein. This phenomenon is typically observed in approximately 75% of cases of ductal adenocarcinoma. The loss of function of this protein enables cell survival and division despite DNA damage, leading to the accumulation of additional anomalies [25,47,64].

Mutations and deletions in *DPC4* (deleted in pancreatic carcinoma, locus 4), *LKB1* (liver kinase B1), and *INK4a* (inhibitor of kinase 4a) are identified in 95% of pancreatic cancer cases. Additionally, the deletion of *MKK4* (mitogen-activated protein kinase 4) is observed in patients with pancreatic cancer. Interestingly, DPC4 triggers metastases despite its absence in pancreatic cancer cells. Furthermore, the mutation of the *LKB1* gene is linked to Peutz-Jeghers syndrome, which is associated with an increased risk of pancreatic cancer [49].

**Figure 3:**
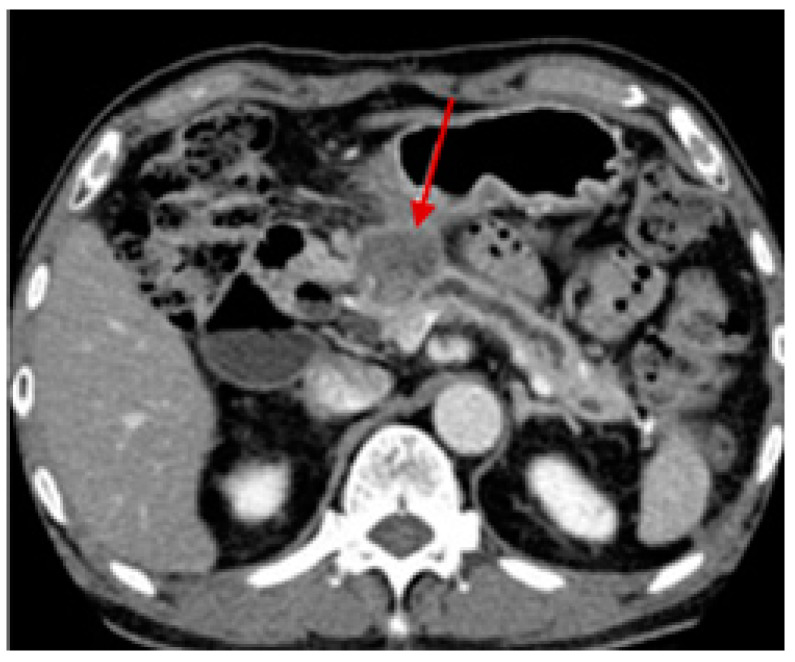
Computed tomography scan of a 73-year-old male patient with pancreatic cancer in the pancreas head with about 3 cm (arrow). Reprinted with permission from [65].

Besides the physical examination, imaging tests are conducted to investigate suspected cancerous areas, assess the tumor localization or metastasis status, analyze treatment efficacy and progress, and monitor for cancer recurrence after treatment. Among the imaging tests, computed tomography allows detailed cross-sectional images to be obtained after the injection of intravenous (IV) contrast and is commonly used in the diagnosis of pancreatic cancer. A biopsy is performed using endoscopic ultrasound to guide the needle to the specific site. Although computed tomography can also be applied (Figure 3), it is not as usual [49,65].

Another imaging test that can examine the pancreatic ducts is called cholangiopancreatography. Its objective is to assess whether the ducts are obstructed, narrowed, or dilated due to the presence of a tumor. Somatostatin receptor scintigraphy can be particularly useful for detecting pancreatic NETs. Positron emission tomography (PET) involves injecting a slightly radioactive form of sugar with an affinity for cancer cells. A specialized camera is used to create an image of areas with radioactivity in the body. This imaging test is sometimes employed to investigate the spread of exocrine pancreatic cancer. However, since NETs grow slowly, they may not appear well in PET examinations [25,66].

Lastly, another imaging test for pancreatic cancer detection is angiography, a test that evaluates blood vessels. This detection technique consists of injecting a contrast dye into an artery to set out the blood vessels, followed by an X-ray, allowing one to visualize if the blood flow in a precise region is blocked or compressed by the existence of a tumor. It also allows checking if pancreatic cancer has increased through the wall of specific blood vessels [66].

Besides imaging tests, it is of utmost importance to perform blood tests for an early cancer diagnosis to determine the most effective treatment. Diverse types of blood tests are performed depending on where the pancreatic cancer is located. For example, for a tumor located in the exocrine part, the hepatic function test, which measures bilirubin levels, is performed. Moreover, for the premature finding and diagnosis of this type of cancer, several tumor markers should also be analyzed in the bloodstream. In this sense, CA 19-9 carbohydrate antigen and the carcinoembryonic antigen (CEA) are the most frequent markers analyzed being approved by the Food and Drug Administration (FDA) for that purpose. However, the American Society of Clinical Oncology does not recommend it in a diagnostic phase since these tumor markers have low sensitivity specificity and can also be found in high levels in people due to other reasons besides pancreatic cancer [67,68].

In the case of pancreatic neuroendocrine tumors, blood tests are conducted to analyze pancreatic hormone levels, namely insulin, gastrin, glucagon, somatostatin, pancreatic polypeptide, and vasoactive intestinal peptide. Furthermore, the levels of chromogranin A (CgA) and glucose and c-peptide (for insulinomas) may also be evaluated [69].

In carcinoid tumors, a blood test can be executed to detect the presence of serotonin, which is produced by many of these tumors. Additionally, urine analysis can evaluate serotonin and related chemicals, such as 5-HIAA and 5-HTP. Although the methods mentioned above may indicate the presence of pancreatic cancer, the only definitive way to confirm it is through a biopsy. This procedure involves obtaining a small tissue sample from the tumor and examining it under a microscope. Biopsies can be performed percutaneously (through the skin), endoscopically (endoscopic), or through surgery (surgical), with the latter being less common [25,49].

## 5. Conventional Treatment of Pancreatic Cancer

Systemic chemotherapy is employed to alleviate symptoms and enhance the survival rate of cancer patients. However, pancreatic cancer tumors exhibit a significant resistance to chemotherapy. Response rates are below 20% for various chemotherapeutic agents, including antimetabolites, alkylating agents, antibiotics, and anthracyclines, whether used individually or in combination therapy [70].

GEM, a pyrimidine antagonist, has shown promising results. This drug substituted 5-fluorouracil as a first-line treatment for metastatic pancreatic cancer due to better overall survival time and greater clinical improvements by alleviating usual symptoms such as pain, functional impairment, and weight loss. But, despite all these results, GEM only increases life expectancy by an average of 6 weeks, ranging from 4.5 to 6 months [71,72].

Nowadays, GEM is used both in monotherapy and combination therapy with other chemotherapeutic agents, namely, fluorouracil, pemetrexed, irinotecan, exatecan, cisplatin, oxaliplatin, paclitaxel, and docetaxel [70]. A phase III clinical trial revealed that by adding oxaliplatin to GEM, there was an increment in the response rate and progression-free survival, providing clinical benefits, although it still failed to improve the survival rate. Another example was the addition of erlotinib to GEM, which increased the 1-year survival rate in comparison to treatment by GEM alone [73,74].

In 2011, FOLFIRONOX^®^, a mixture of 5-FU, leucovorin/folinic acid, oxaliplatin, and irinotecan, revealed a superior survival rate in patients with pancreatic cancer, compared to GEM as the sole agent, which led to the use of this drug as therapy of choice in this type of cancer. Nevertheless, the toxicity profile of the drug was not insignificant, showing an elevated risk of myelosuppression, fatigue, vomiting, diarrhea, and thrombocytopenia. Despite the improvement in overall survival rate, it remained low, highlighting the need for more effective treatments in the management of pancreatic cancer [70,75].

Radiotherapy is frequently consumed in combination with systemic chemotherapy, as it does not provide significant benefits when used alone after pancreatic cancer surgery. However, a randomized phase III study demonstrated that patients derived greater benefits from the combination of chemotherapy and radiotherapy compared to chemotherapy alone in isolation [76,77].

Radiotherapy offers significant benefits in terms of local control and can improve the resectability rate after downstaging. However, it does not lead to notable improvements in mean survival rates for patients with non-resectable pancreatic cancer. The introduction of a novel and specific technique known as stereotactic radiotherapy, which delivers targeted radiation doses to tumors using imaging guidance, has shown some improvements in survival outcomes. Nevertheless, the overall survival rate for pancreatic cancer patients remains unsatisfactory, and there are concerns about the associated treatment toxicity. Further studies are necessary to determine the role of radiotherapy in resectable pancreatic tumors [78].

Somatostatin and its analogs, peptide hormones, can act as inhibitors of tumor cell growth by triggering signal transduction, which negatively controls cell growth, or through downregulation of tumor growth [79]. Szende et al. provided evidence that somatostatin can trigger tumor regression through a cell death program mechanism [80]. However, somatostatin monotherapy does not offer therapeutic benefits in the treatment of pancreatic cancer. Ebert et al. demonstrated that the use of a somatostatin analog called octreotide at a high dose resulted in a median survival of 6 months for patients with advanced pancreatic cancer, whereas the low dose only achieved half of that survival time [81,82].

Besides somatostatin and its analogs, estrogens can be potential candidates for the treatment of pancreatic cancer, given the presence of estrogen receptors in pancreatic carcinomas. Rosenberg et al. described that patients receiving a combined regimen of octreotide and tamoxifen exhibited a significant average survival benefit of 12 months as compared to those undergoing monotherapy [83,84].

The use of leuprolide (Lupron), a luteinizing hormone agonist, by itself or combined with somatostatin, demonstrated in vitro and in vivo activity in hamsters with pancreatic cancer. Zaniboni et al. performed a phase II clinical trial evaluating the leuprolide-tamoxifen combination, but the results were disappointing, achieving only a mean survival of 5 months. In short, the impact of hormone therapy on this type of cancer is quite limited [85,86,87]. The application of this therapy has been increasing, particularly in pancreatic cancer. The microenvironmental immunosuppressive tumor in pancreatic cancer plays a crucial role in disease progression and is linked to the limited effectiveness of conventional therapies. Typically, the microenvironment in pancreatic cancer comprises a fibrotic stroma with significant stromal density, acting as a barrier that hinders the delivery of cytotoxic drugs and limits the access of T cells to tumor cells [88,89]. Furthermore, the extensive infiltration of myeloid cells, including macrophages and immature/suppressor myelogenous cells derived from myeloid cell derivatives (MDSCs), accumulated during the progression of pancreatic cancer can induce T cell dysfunction. Consequently, depleting macrophages or MDSCs enhances the infiltration and activation of CD8^+^ T cells, thereby improving the immune response against tumor cells [89,90].

According to Zhang et al. myeloid cells play a crucial role at various stages of pancreatic carcinogenesis, and thus, depleting myeloid cells during the development of pancreatic cancer can hinder tumor formation [90]. In their study, they also found that myeloid cells act as regulators of immune checkpoint ligand PD-L1 expression in tumor cells through the activation of epidermal growth factor/mitogen-activated protein kinase signaling. PD-L1 is an immune-inhibitory molecule that suppresses T-cell activation, contributing to tumor progression. Consequently, inhibiting MAPK can enhance tumor susceptibility to PD-1/PD-L1 blockade, presenting a potential new therapeutic strategy for treating pancreatic cancer [89,90].

Lastly, the other option for the management of pancreatic cancer is surgery. Nowadays, there are two types of surgery for pancreatic cancer: a potentially curative surgery, which is performed when diagnostic test results show that removal of the entire tumor is possible, and palliative surgery, which can be executed if diagnostic tests show that the tumor is already too metastasized to be fully removed, only being performed to alleviate symptoms or to prevent future complications such as blockage of the bile duct or intestine.

However, since most pancreatic cancers are only diagnosed at stage IV, since there are no noticeable symptoms in the first stages, and metastases already exist, resection surgery is not usually performed [70].

Besides the adverse effects, conventional therapies are constrained by delivery problems, compromising their efficacy. Thus, it is important to optimize a delivery strategy for therapeutic agents, namely using nanotechnologies. These allow the transport of therapeutics selectively into tumor tissue, minimizing toxicity in healthy tissues and reducing collateral events and resistance related to the immune system [91].

## 6. Advantages and Disadvantages of Nano-Based Drug Delivery Systems

Nanomedicine is defined as the application of nanobiotechnology to medicine based on the use of materials and devices at the nanoscale for drug delivery. In addition to these applications, nanobiotechnology is also being developed for the implementation of nanosurgery. Such technological advances can be useful for nanoscale systems administration in the human body and for the treatment and diagnosis of health problems [92].

Applying nanomedicine to the treatment of pancreatic cancer has numerous advantages when compared to conventional therapies, such as controlled and sustained release of drugs, lower systemic toxicity since the delivery of the drug is directed through the conjugation of ligands on the surface of the nanoparticles; reduced number of administrations, as there is an increase in drug time and concentration at the local level; overcome tumor barriers; higher penetration in the tumor microenvironment; passive accumulation in tumors due to permeability and retention effect (EPR) and better stability and anticancer activity [93,94].

In addition to these advantages, since pancreatic cancer is highly metastatic, advances in knowledge of tumor progression and nanomedicine make it possible to slow down tumor metastatic progression. The targeting of distinct stages of cancer metastasis makes it possible to act on several aspects of the metastatic cycle. Thus, among the strategies described to act on metastatic progression is the targeting of cancer stem cells because they are the starting point for the development of metastases. Also, the remodeling of the microenvironment within the tumor microenvironment is necessary because it is the area where the tumor cells, stroma cells, and secreted factors communicate among themselves and where the tumor mass develops. The tumor site remodeling improves drug delivery and nanoparticle distribution. Moreover, in this type of cancer, the EPR effect is not effective for nanoparticle penetration due to the dense stroma-rich solid tumor. Lastly, targeting oncogenic exosomes would decrease niche formation and prevent metastasis [95,96].

Nanomedicine thus enables the circumvention of many obstacles related to conventional therapies, thus increasing the effectiveness of treatment as well as allowing the exploration of alternative therapies [97]. Nanocarriers are 100 to 10,000 times smaller than human cells. They can maintain stability in physiological environments and passively target pancreatic tumor cells due to the enhanced EPR effect. Their size allows them to leak out of blood vessels and bind to carcinoma cells [19]. However, nanoparticles come with a set of challenges. Their non-physiological chemical surfaces can lead to non-specific cellular interactions, potentially resulting in precipitation and subsequent cellular damage. Systemic actions of certain systems, as opposed to localized ones, might introduce adverse effects. Furthermore, some excipients used in their formulation may possess toxicity for the human body [97].

Nanoparticles made from polymers or natural materials, like modified polyesters, polysaccharides, and proteins (e.g., albumin), offer multifunctional attributes and hold significant potential for localized chemotherapy. They can be designed to actively target tumor cells by pairing with specific recognition elements, such as monoclonal antibodies, which are already employed in oncological treatments [98]. In addition, nanoparticles can be customized or tailored to be selectively directed at a particular target, or their path can be monitored. Thus, specific functional groups can be incorporated that aid in targeting, or characteristics such as fluorescence can be altered to track their journey after administration to their target [91].

The nanostructured materials are processed from crude nanomaterials that provide specific shapes or with certain predefined functionality and which are divided into polymers, such as dendrimers, micelles, etc., and non-polymers, such as carbon nanotubes, metal nanoparticles, among others [98].

Several works describe the use of nanotechnology to study the potential use of numerous drugs that have shown promise but have not yet been used due to their inability to be properly administered, and many of these problems are related to their low solubility. Among them are efforts to optimize formulations of GEM (currently the first-line treatment) as well as to improve its efficacy [99]. GEM has been widely studied and tested in nanosystems based on polymer-drug conjugate, mixed micelles, dendrimers, albumin, and inorganic nanoparticles [91].

Paclitaxel, an antimicrobial chemotherapeutic agent used for solid tumors, including pancreatic cancer, has limited efficacy because of its poor water solubility. It has been explored in nanosystems such as mixed micelles, ultrasound-responsive nano-emulsions, and albumin-based systems [100,101].

It was also approved in 2013 by the FDA for the medicine Abraxane^®^, a nanoparticle-albumin-bound paclitaxel for the treatment of metastatic pancreatic cancer. This drug showed therapeutic advantages when administered in combination, namely by increasing the intratumoral concentration of GEM, partially due to the EPR effect, thus potentiating the effect of this drug. In addition, combination therapy has shown significant improvements in survival in patients with pancreatic cancer [102,103,104].

Thus, in the next sections, different nanostructures will be described that are being used as delivery vehicles for one or more drugs to increase their half-life, stability, and accumulation at their target site.

## 7. Nano-Based Drug Delivery Systems for Pancreatic Cancer Treatment

In recent years, significant work has been carried out to improve not only the quality of life but also to extend the life span of patients with pancreatic cancer. There are numerous nano-based formulations with chemotherapeutic agents already known in distinct phases of clinical trials, and some of them are already in phase III, close to being approved and marketed. Among them there are the nanoparticles albumin-bound paclitaxel, the pathotropic nanoparticles gene delivery, the micelle nanoparticles, and the liposomal nanoparticles [72]. For example, Rexin-G consists of a pathotropic retroviral-based nanoparticle/gene delivery vector constructed from the co-transfection of human embryonic kidney 293T cells with Moloney murine leukemia virus. This system encodes a dominant-negative mutant construct of the human G1 cyclin gene and was evaluated for the first time in the treatment of pancreatic cancer in the Philippines by Gordon et al. It showed tumor stabilization and arrest of tumor growth in three of three patients with no experience of dose-limiting toxicity [105].

The future of pancreatic cancer treatment also undergoes interference-based RNA (iRNAs) therapy. This therapy is progressing, proving to be a viable alternative to conventional treatments, especially regarding specificity, toxicity, and the overcoming of existing resistance to numerous drugs. iRNA therapy is an endogenous process that acts by silencing the genes that can cause any type of degradation of the mRNA once the RNA sequence used is known. siRNAs have been assigned to ensure propagation both in vitro and in vivo. siRNAs are involved in the silencing of post-transcription genes for specific mRNAs. The RNA-induced silencing complex acts as a guide to cleave mRNAs through a sequence complementary to siRNA and can be recycled repeatedly to cleave additional mRNAs [106,107]. It is important to note that incorporating chemically synthesized siRNAs into the cell may cause the activation of the previously described process, which may be useful as gene therapy to suppress specific genes associated with various pathologies. On the other hand, the vectorization of siRNA into the target cell is still an obstacle to this innovative therapy, mainly due to the administration of siRNA in vivo, due to its lack of stability and vulnerability to degradation, and its highly anionic charge [108]. However, nanoparticles in the form of liposomes, lipid polymers, and dendrimers may be the key to this problem. These delivery systems can encapsulate the siRNA, protecting it from degradation and elimination by the immune system, as well as delivering it to the specific target cell [109,110,111].

Moreover, these nanoparticles offer promising opportunities for both direct and indirect immunomodulation in pancreatic cancer. Direct immunomodulation concerns engineering nanoparticles to directly interact with immune cells, influencing their activation and function. These nanoparticles act as carriers for immune-stimulating agents, such as cytokines or antigens, enhancing the immune response against pancreatic cancer cells. On the other hand, nanoparticles can indirectly modulate the immune system by targeting the tumor microenvironment. These systems can deliver therapeutic molecules to reprogram immunosuppressive cells, like dendritic cells, T cells, and antibody receptors, and reduce immune checkpoints, leading to enhanced antitumor immune responses. Also, nanoparticles can facilitate the delivery of immunomodulatory agents, such as immune checkpoint inhibitors, to specific sites, improving their efficacy while minimizing target effects. Nanoparticles can also be conjugated with some molecules in order to target specific targets in the tissues and enhance their effect. So, by harnessing the potential of nanotechnology, innovative strategies for immunomodulation hold the promise of revolutionizing the treatment landscape for pancreatic cancer [15,112].

In these sections, the several types of nano-based drug delivery systems used for pancreatic cancer treatment are fully disclosed. In Table 3, there is an overview of several examples of nanocarriers applied in this cancer treatment, which will be then described.

### 7.1. Lipid-Based Nanoparticles

**Figure 4:**
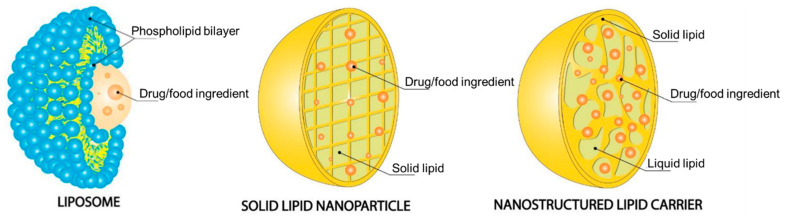
Structural features of liposomes, solid lipid nanoparticles, and nanostructured lipid carriers. Reprinted with permission from [138].

Among the various lipid-based nanocarriers, the most widely used are liposomes, which are extensively studied. Liposomes (Figure 4) are colloidal spherical structures that enable specific targeting flexibility and permit the delivery of both insoluble and water-soluble compounds. However, due to some disadvantages such as their low stability, especially during long-term storage, in aqueous dispersions, high cost, low payload, and fast release, they are not the only type of lipid-based nanoparticles used, and other types are also coming to the frontline. Solid lipid nanoparticles (SLN) (Figure 4) were developed as an alternative to liposomes. They are composed of a solid lipid or mixture of different solid lipids at room and body temperature, and therefore, they present better stability and lower release rate when compared to liposomes. However, SLNs have a set of drawbacks, such as low encapsulating capacity when it comes to bioactive compounds and a high water content, which make them unsuitable nanocarriers for food ingredients. To solve these problems, NLC (Figure 4) was developed, whereas the main difference, when compared to SLN, is regarding the lipid composition. NLCs are made of a mixture of solid lipid and liquid lipid, which, due to the resulting imperfect structure, allows a higher loading capacity and a controlled release rate [138].

#### 7.1.1. Liposomes

Liposomes are small spherical particles containing one or more phospholipid bilayers with an internal hydrophilic compartment through the introduction of phospholipids in an aqueous solution. Depending on the design, they can vary between 10 nm and several micrometers. The application of liposomes for delivering drugs has had a significant impact on medicine. Drugs with high toxicity or low bioavailability benefited from the stabilization and biodistribution added by liposomes [81,139]. A study in which the efficacy and safety of nanoparticles of liposomal cisplatin (lipoplatin) conjugated with GEM in patients with pancreatic refractory cancer were established demonstrated that there was a 50% reduction in the sum of products of the diameters perpendicular lesions lasting at least four weeks and that the mean survival from the start of treatment was four months. The bi-weekly dose of treatment was 100 mg/m^2^ of lipoplatin and 1000 mg/m^2^ of GEM and was well tolerated without evidence of neurotoxicity or nephrotoxicity. A liposomal formulation containing Raf-1 antisense oligonucleotides also demonstrated improved antitumor activity against human pancreatic tumors in an athymic mouse model [140].

Currently, nanoparticles conjugated to antibodies or peptides to deliver the siRNA to target cancer cells have been the goal of many researchers [118]. The transferrin receptor (TfR), folate receptor, and Arginine-Glycine-Aspartic ligands are several examples of cell surface target receptors used since several studies prove that they are highly expressed in various types of tumors [141,142]. Pirollo et al. used liposomes that contained a TfR antibody conjugated to its surface, carrying siRNA against HER-2. They compared the siRNA delivery system with and without TfR antibody and concluded that there was a superior delivery of siRNA in pancreatic tumors in a murine xenograft model in which TfR antibody was contained (*p* ≤ 0.002). In addition, they also showed that lipocomplex-siRNA could inhibit HER-2 expression in pancreatic tumor cells and increase sensitivity to GEM, a promising advance in the treatment of pancreatic cancer [113].

In 2015, Adiseshaiah et al. designed a nanoliposomal system (MM-398) in which they encapsulated irinotecan (topoisomerase I inhibitor). This formulation was permitted for use in combination therapy with 5-fluorouracil and folinic acid in the second-line therapy of patients with metastatic pancreatic ductal adenocarcinoma after previous gemcitabine-based therapy. This combination increased intratumoral deposition of the irinotecan drug, showing a significant improvement in overall patient survival compared to therapy alone with 5-fluorouracil or folinic acid [14,15,16,17,18,19,20,21,22,23,24,25,26,27,28,29,30,31,32,33,34,35,36,37,38,39,40,41,42,43,44,45,46,47,48,49,50,51,52,53,54,55,56,57,58,59,60,61,62,63,64,65,66,67,68,69,70,71,72,73,74,75,76,77,78,79,80,81,82,83,84,85,86,87,88,89,90,91,92,93,94,95,96,97,98,99,100,101,102,103,104,105,106,107,108,109,110,111,112,113,114,115,116].

A common goal of using liposomes is to improve the stability and target-specific delivery of a therapeutic drug. In this sense, Matsumoto et al. developed a strategy for the delivery of GEM to pancreatic cancer cell lines by encapsulating it into a liposome named FF-10832 and administrating it to mice. It was concluded that when using FF-10832, the long-term stability of GEM circulating in plasma was enhanced when compared to the free form (*p* < 0.001). Also, in mice with Capan-1, SUIT-2, and BxPC-3 tumors, FF-1083 exhibited better antitumor activity when contrasted to the GEM-free form. Therefore, FF-10832 shows great promise to be applied for pancreatic cancer treatment [117].

In 2019, Zinger et al. showed the importance of remodeling the extracellular matrix to improve drug delivery by surpassing the dense stroma of pancreatic adenocarcinomas. In this work, they used collagenase-loaded liposomes as a pre-treatment to disassemble the dense extracellular collagen stroma matrix, then a subsequent treatment with paclitaxel-loaded micelles. The results showed an increased drug penetration into the pancreas and improved effectiveness of treatment, also proving to be an encouraging strategy for the treatment of other types of cancer [143].

**Figure 5:**
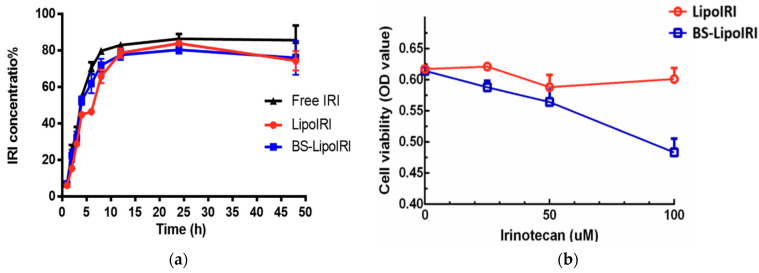
Profile of irinotecan (IRI) in vitro release from LipoIRI and BS-LipoIRI (**a**); in vitro cytotoxicity of LipoIRI and BS-LipoIRI for BxPC3 pancreatic tumor cells (**b**). Adapted from with permission [118].

More recently, in 2022, Lin et al. developed an anti-epidermal growth factor receptor (anti-EGFR) and anti-fibroblast activation protein bispecific antibody-targeted liposomal irinotecan (BS-LipoIRI) that was able to target and bind to pancreatic tumor cells and tumor-associated fibroblasts that are linked with tumor proliferation and aggregation. A drug encapsulation efficiency of 80.95% was achieved for BS-LipoIRI, a drug loading of 8.41%, along with a particle size of 125 nm. The results also showed a similar release rate and a better cellular uptake efficacy of BS-LipoIRI when compared to LipoIRI alone. In vivo testing results also concluded that BS-LipoIRI inhibited cancer growth up to 46.2% when contrasting to the controls, showing the great promise of BS-LipoIRI as a possible novel treatment for pancreatic cancer (Figure 5) [118].

Liposomes have proven to be valuable in drug delivery for pancreatic cancer treatment, enhancing drug stability and target-specific delivery. For example, the conjugation of liposomes with targeting agents like transferrin receptor antibodies has demonstrated superior siRNA delivery to pancreatic tumors and increased sensitivity to chemotherapy. Strategies involving collagenase-loaded liposomes to remodel the extracellular matrix for better drug penetration have also been explored. However, liposomes have shown some drawbacks, including stability issues such as sedimentation, aggregation, and coalescence over time, leading to reduced shelf-life and changes in size. This instability affects reproducibility, encapsulation efficiency, and drug release during storage. So, controlling their stability during and after production is crucial. Liposomes may also accumulate in liver and splenic macrophages, causing splenomegaly and hepatotoxicity. Consequently, due to these limitations and other disadvantages, liposomes are not considered robust enough for drug delivery. So, these issues led to the development of other lipid-based nanocarriers [144,145].

#### 7.1.2. Solid Lipid Nanoparticles

SLN offers immense potential as a novel drug delivery platform by improving the therapeutic effectiveness and safety profile of current treatments. Their size typically ranges from 100 to 700 nm, and they can penetrate and accumulate within the tumor cells [144,146].

In recent years, numerous studies have been made demonstrating these nanocarriers’ effectiveness in delivering various chemotherapeutics drugs to the tumor site in comparison to the free drug treatment in human studies. Sutaria et al. evaluated the usage of a combination of aspirin, curcumin, and free sulforaphane encapsulated in SLN for the chemoprevention of pancreatic cancer. The SLN, one for the encapsulation of aspirin and another for the encapsulation of curcumin, were formulated by a modified solvent evaporation method, which resulted in nanocarriers with an average particle size of 150 and 250 nm and encapsulation efficiency of 85% and 69%, respectively. The cell viability and apoptosis of pancreatic cancer cell lines, namely human pancreatic cancer cell lines MIA PaCa-2 and Panc-1, were also evaluated. These studies showed that with a low concentration of aspirin-loaded SLN (25 µM), curcumin-loaded SLN (2.5 µM), and free sulforaphane (5 µM), the viability of MIAPaca-2 cells was reduced by 43.6% and Panc-1 cell lines by 48.49% (*p* < 0.0001), with apoptosis values of 61.3% for MIAPaca-2 cell lines and 60.37% for Panc-1 cell lines in comparison to the results obtained for the administration of the free-form of the drugs [120].

Affram et al. investigated the cytotoxic effect of GEM encapsulated in different SLN structures on pancreatic cancer cell lines (MiaPaCa-2) and primary pancreatic cell lines (PPCL-46). Glyceryl monostearate, polysorbate 80, and poloxamer 188 were used to produce the distinct SLN structures. In vitro studies showed a higher cytotoxic effect (*p* < 0.001) on the primary pancreatic cancer cell lines (IC_50_(2D) = 27 ± 5 μM; IC_50_(3D) = 66 ± 2 μM), in comparison to the free form of GEM (IC_50_(2D) = 126 ± 3 μM; IC_50_(3D) = 241 ± 3 μM). A similar result was observed for the MiaPaCa-2 pancreatic cell lines (*p* < 0.026), being the inhibition values for GEM-loaded SLN of IC_50_(2D) = 56 ± 16Μm and IC_50_(3D) = 127 ± 4 μM, whereas, for free GEM, the values achieved were IC_50_(2D) = 188 ± 46 μM and IC_50_(3D) = 254 ± 52 μM. Therefore, SLN improved the therapeutical effect of GEM on pancreatic cancer cell lines [119]. Thus, these two studies demonstrated the potential of using SLN as novel carriers for drug delivery to treat pancreatic cancer.

Overall, SLNs are versatile drug delivery systems for diverse routes, improving therapeutic effectiveness and safety profiles of treatments for pancreatic cancer. For example, GEM-loaded SLNs exhibited higher inhibition values on both primary pancreatic cancer cell lines and MiaPaCa-2 pancreatic cell lines. Also, their structure allows them to be finely tuned based on active ingredients and excipients. However, modified release depends on the particle’s solid state (e.g., crystallization and physicochemical transitions). Lipidic materials, typical components of SLNs, undergo physicochemical transitions, leading to a denser and arranged matrix, but also an altered shape that may not be favorable for drug molecules and lead to unwanted drug release during storage. Moreover, poor drug payload may occur due to retarded or suppressed crystallization. In this sense, the use of spatially different molecules is needed in order to overcome these drawbacks [144,146].

#### 7.1.3. Nanostructured Lipid Carriers

NLCs are considered second-generation lipid nanoparticles and show a spherical structure with a mixed solid and liquid matrix loading the drug. These systems present better entrapment efficiency, loading efficiency, and stability than the previous SLN. NLC comes to overcome some SLN limitations [144,146,147].

NLC is obtained by mixing solid lipids with incompatible liquid lipids, leading to special structures of the lipid matrix, which increases the imperfections on the matrix and leads to a higher loading capacity while maintaining the stability of the formulation. These formulations allow for circumventing the limitations associated with SLN, like low drug loading capacity, dosage adjustment of the drug release profile, and possible drug expulsion during storage [148,149,150].

There are several works in which these nanoparticles have been widely used. It has also been emerging as an ideal drug delivery platform for the pharmaceutical market for oral or parenteral administration and delivery of nucleic acids such as miRNA for tumor genes. The advantages of these nanoparticles concerning the SLN and their superior biocompatibility, high biodegradability, and low immunogenicity make them useful for many clinical applications [151,152,153,154,155].

Pramanik et al. synthesized a lipid-based nanoparticle for systemic delivery of microRNA (miRNA) expression vectors to cancer cells (nanovector). The chosen miRNAs are known to be downregulated in most pancreatic cancers and were loaded into cationic NLC. The safety profile in using these systems to deliver miR-34a or miR-143/145 to treat the pancreatic cancer xenograft model was evaluated. After blood injection, it was concluded that the miRNA-loaded cationic NLCs interrupted pancreatic cancer progression in MiaPaCa-2 subcutaneous xenografts (*p* < 0.01 for miR-34a; *p* < 0.05 for miR-143/145) and reduced tumor cell proliferation. Furthermore, no histopathological changes or biochemical toxicity were seen in mouse animal models, thus revealing the safety of cationic NLCs when applied in vivo [121].

Later, Zhihe et al. analyzed the pancreatic antitumor activity of hyaluronic acid-coated, prodrug-based NLC for the release of GEM and Baicalein (BCL). They developed NLC coated outside with hyaluronic acid (HA) on the surface, which has the ability to bind the upregulated receptors in numerous cancer cells, and GEM and BCL were loaded in the core of NLC. GEM-stearic acid lipid prodrug (GEM-SA) and hyaluronic acid-amino acid-baicalein (HA-AA-BCL) were produced. The combination of these prodrugs yields the system HA-GEM-BCL NLCs, synthesized by nanoprecipitation technique and allowed to mark the cancer cells and co-deliver the drugs. In the in vitro cytotoxic studies on human pancreatic adenocarcinoma cell lines (AsPC1 cells), NLCs could successfully target and enter the cancer cells upregulated HA receptors and showed cytotoxicity activity on tumor cells. Regarding the in vivo study, NLCs showed a remarkable tumor growth inhibition capacity in the murine pancreatic cancer model. This system enhances the synergistic effect of this co-delivery for anti-pancreatic cancer drugs, improves the targeted anti-tumor efficacy, and brings a new potential delivery system for this disease [122].

In summary, NLCs were developed to overcome SLN limitations, namely low drug loading and drug expulsion during storage. By adding a liquid lipid to the solid matrix, NLCs increase loading capacity and maintain formulation stability. Thus, the papers mentioned above demonstrate the potential of using NLCs as a potential drug delivery platform for the treatment of pancreatic cancer.

### 7.2. Hybrid Nanoparticles

With the emerging siRNA-based therapies, it has also emerged the application of hybrid nanoparticles as nanocarriers for siRNA delivery. Hybrid nanoparticles, also called lipid-polymer hybrid nanoparticles, are composed of a single layer or a bilayer lipid shell surrounding a polymeric core and theoretically combine the advantages of lipid and polymeric nanoparticles. The cationic polymeric core encapsulates the drug and the siRNA, while the lipid layer offers a protective effect and excellent biocompatibility and stability [123]. A study made in 2006 reported the therapeutic potential of the nanoparticles complexed with siRNA. In this study, the authors used PEG—(poly (ethylene glycol))—modified liposomes and contained on their surface-active targeting moieties to improve tumor bioavailability. These modified liposomes delivered apoprotein B siRNA to non-human primates. The single administration at the systemic level resulted in a significant reduction in mRNA and apoprotein B (apo B) expression, as well as a reduction in total cholesterol. In this study, it was also determined that the complex formed by the modified liposome and siRNA was non-toxic and allowed the sustained blockade of apo B for 11 days [116].

In 2015, Zhao et al. explored the application of a lipid-polymer hybrid nanoparticle to co-deliver GEM and HIF1a siRNA (si-HIF1a), in which factor HIF1a increased the drug resistance of GEM for pancreatic cancer treatment. By coating the polymer core with a PEGylated lipid bilayer, nanoparticle aggregation was prevented, si-HIF1a did not degrade in the serum, and no GEM leakage was observed. Therefore, the lifespan of the drug in the bloodstream was prolonged, and drug release improved. Si-HIF1a was effectively absorbed by the nanocarrier, which also showed high-loading contents of GEM. Most importantly, these lipid-polymer hybrid nanoparticles could effectively deliver GEM and si-HIF1a to the tumor cells and suppress HIF1a expression both in vivo and in vitro studies and presented an incredible capacity for inhibition of tumor metastasis in orthotopic tumor models [123]. The results of these studies highlight the promising therapeutic application of nanoparticle-siRNA complexes in the treatment of human diseases [156].

The advantage of using cationic lipid polymers to deliver siRNA stems from their ability to traverse the cell membrane, promote leakage of the endosomal compartment, and target genes while keeping biocompatibility. However, there are also drawbacks, such as the induction of a response by interferon, and undesired interactions may occur with negatively charged serum proteins. Cationic lipid polymers can interact with negatively charged siRNA through ionic interactions. Thus, the self-assembled lipoplexes confer protection to the siRNA, preventing it from undergoing enzymatic degradation, increasing its cellular uptake by endocytosis, increasing the release of siRNA from the endosomal/lysosomal compartment, and promoting its accumulation in the cytosol [118].

Currently, there are various formulations commercially available with cationic lipid polymers, such as Lipofectin^®^, Lipofectamine^®^ (Invitrogen, Waltham, MA, USA), Dharmafect^®^ (Dharmacon^®^, Lafayette, CO, USA), RNAifect^®^ (Qiagen, Hilden, Germany), and TransIT TKO^®^ (Mirus, Madison, WI, USA). These formulations were studied as transfection reagents for in vitro administration of siRNA, and it was concluded that the lipid/siRNA ratio affects the colloidal properties of lipoplexes (size and zeta potential), facilitates the cellular internalization of lipoplexes, and dissociates nucleic acids in the cytosol [157].

Besides siRNA-based therapies, hybrid nanoparticles have been used to develop novel treatments for pancreatic cancer. In 2020, Yu et al. developed a technique where they encapsulated paclitaxel in an albumin nanoparticle and then integrated it into a modified thermosensitive liposome, aiming to improve drug penetration into pancreatic ductal adenocarcinoma. This type of cancer is known for its dense tumor stroma, which poses a significant challenge for drug delivery. By incorporating paclitaxel into the liposome, the drug’s retention in the solid tumor was enhanced. Moreover, a photothermal agent, IR-780, was added. When photothermal therapy was combined with nanoparticle administration, the IR-780 widened the tumor’s interstitial space, promoting the release of the drug system encapsulated within the modified liposomes [124].

Overall, hybrid nanoparticles have improved pancreatic cancer outcomes by enhancing drug penetration through the dense tumor stroma while the lipid-polymer layer increases the protection and stability of the drug. Commercial formulations of cationic lipid polymers have also shown potential as siRNA transfection agents and encapsulating paclitaxel and a photothermal agent enhanced drug release and treatment efficacy. All these findings highlight the potential of hybrid nanoparticles for targeted cancer therapy.

### 7.3. Polymer Nanoparticles

Nanoparticles based on natural polymers are usually biocompatible, biodegradable, and non-toxic, although there are often stability problems when delivered. Within natural polymers, chitosan, gelatin, albumin, and alginate are the most used. However, synthetic polymers may also be used in the form of a preformed polymer, and polyesters such as polycaprolactone (PCL), poly (lactic acid) (PLA), or monomers can be polymerized in situ. There are a few advantages to using such nanoparticles in drug delivery, such as biocompatibility, increased drug stability, readily manufactured in large quantities, non-immunogenicity, and non-toxicity, among others [158].

**Figure 6:**
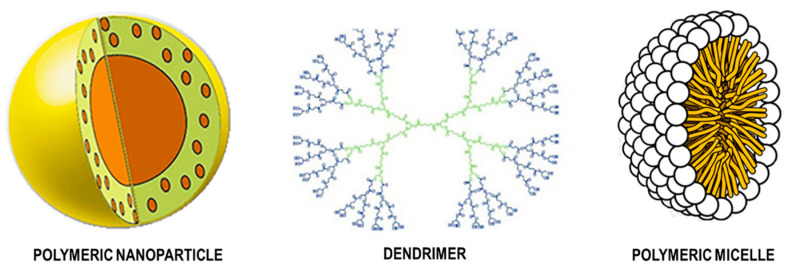
Structural features of polymeric nanoparticle, dendrimer, and polymeric micelle. Adapted with permission from [160,161].

Polymeric nanoparticles can be vesicular systems (nanocapsules) or matrix systems (nanospheres). Nanocapsules are systems in which the drug is confined to a cavity surrounded by a single polymer membrane. Nanospheres are systems in which the drug is dispensed by a polymer matrix. Within polymer nanostructures beyond polymer nanoparticles, there are dendrimers, micelles, and conjugated drugs (Figure 6) [159,160,161].

#### 7.3.1. Natural Polymeric Nanoparticles

Albumin is a carrier protein with a broad interest in drug delivery as it is biocompatible, biodegradable, non-toxic, and non-immunogenic. Many authors consider it an ideal material for producing nanoparticles for drug delivery. The accumulation of albumin nanoparticles can occur either by passive (active EPR) or active segmentation due to the high affinity with cellular receptors overexpressed in tumor cells [162].

Significant amounts of the drug can be encapsulated in its matrix since there is a high number of binding sites present in the albumin molecule. This protein can also improve the solubility of lipophilic drugs. The conjugation between a compatible and specific ligand and the surface of the albumin nanoparticle confers active cleavage and enables target delivery to a receptor present in the target cells by covalent attachment [163]. A phase I/II study for pancreatic cancer demonstrated the maximum tolerated dose for Nab-paclitaxel (paclitaxel-linked albumin nanoparticles) combined with GEM. The authors also reported a better overall survival (12.2 months) compared to GEM alone [164]. A phase III clinical trial for metastatic pancreatic adenocarcinoma showed that the combination of Nab-paclitaxel with gemcitabine (GEM) not only significantly extended the average survival to 8.5 months, compared to 6.7 months in patients treated with GEM alone but also notably reduced side effects like neuropathy and neutropenia. This improvement is attributed to the chromophore used to dissolve paclitaxel, enabling a higher administered dose of paclitaxel [165].

In another study, multifunctional albumin nanospheres (called C225-GEM/MANs) were manufactured, where albumin acted like an outer shell while the inner core imprisoned magnetic nanoparticles of Fe_3_O_4_ conjugated to GEM. The anti-EGFR (anti-epidermal growth factor receptor-1) and C225 functioned as active agents that directed the delivery system to the target site [166].

Gelatin nanoparticles have also been used. Sing et al. formulated type B gelatine nanoparticles as a targeted nanocarrier for intravenous delivery of gemcitabine therapy in pancreatic cancer. These nanoparticles were also marked with a redox-responsive EGFR. The drug was encapsulated and attached to the polymer backbone using disulfide bridges during the nanoparticle gelation process, which was induced by the ethanol desolvation method. The nanoparticle surface was then covered with a complementary EGFR targeting peptide to provide target specificity or with PEG chains to improve the circulation time. In vitro studies in Panc-1 human PDA cells revealed that gemcitabine encapsulated in EGFR-targeted gelatin nanoparticles and released through disulfide bond cleavage had a significantly improved cytotoxic profile. Furthermore, the in vivo anticancer efficacy was evaluated using an orthotopic pancreatic cancer model. The results confirmed that gemcitabine delivery to the tumor site was notably more efficient when administered through EGFR-targeted gelatin nanoparticles compared to the drug in its solution form. This method offers a significant therapeutic advantage [125].

Chitosan is also a widely studied natural polymer used for formulating polymeric nanoparticles, including in nano-based treatments for pancreatic cancer, due to being non-toxic, possessing high biocompatibility, and being biodegradable. Xiao et al. developed a novel nanobioconjugate, chitosan-based, to deliver GEM and anti-EGFR antibodies into pancreatic tumor cells. EGFR high expression is associated with erroneous development and unrestricted proliferation in various malignant tumors, including pancreatic cancer. The encapsulation rate obtained was 91.63%, with a drug loading of 9.97%. Cell proliferation, colony formation, migration, and invasion in SW1990 cells were drastically reduced [16]. Another study by David et al. formulated chitosan nanocarriers to co-deliver quercetin and 5-fluorouracil. The nanocarrier exhibits an entrapment efficiency for quercetin and 5-fluorouracil of 95% and 75%, respectively. In the in vitro study performed, it was observed high toxicity levels against pancreatic cancer cells, both in 2D and in 3D cultures [126]. These studies demonstrate the potential of chitosan-based nanoparticles for pancreatic cancer therapies.

#### 7.3.2. Synthetic Polymeric Nanoparticles

Micelles are an example of synthetic polymeric nanoparticles. They may be composed of PEG as the hydrophilic component and modified polyaspartate as the hydrophobic component. In this case, the drug binds to the hydrophobic component. Paclitaxel has limited efficacy due to its poor water solubility. Using a micelle formulation, it was possible to overcome this limitation of paclitaxel by encapsulating it into water-soluble micelles. This study showed that with this formulation, it was possible to achieve improved antitumor activity due to the EFR effect [167].

Hamaguchi et al. conducted a phase I clinical trial to obtain the maximum tolerated dose, dose-related toxicity, and pharmacokinetics of NK105, a micellar carrier system for paclitaxel delivery. Pancreatic cancer patients receiving intravenous administration of NK105 were recruited, and the results were encouraging. The patients tolerated this system well, and no one demonstrated significant hematological toxicity. An incomplete response was noticed in a patient with metastatic pancreatic cancer, who received 150 mg/m^2^, but only in hepatic metastases (reduction in the size of 90%), but the impact on the pancreas was not reported specifically [168].

Polymeric micelles can also be modified to enhance specific target efficiency drug accumulation and to facilitate drug release. Tian et al. studied the targeting efficiency of an aptamer-modified micelle carrying the chemotherapeutic drug doxorubicin (DOX). The same group had already shown in previous studies the capacity of an aptamer to guide DOX to accumulate in the tumor tissue. After the in vitro cytotoxicity studies and laser confocal microscopy, the group concluded that the aptamer-modified micelles improved the targeting and cytotoxicity effect towards human pancreatic tumor cells (Panc-1 cells) when compared to the free form of DOX and not-modified micelles. This new delivery system showed the best penetration into the three-dimensional spheroid of Panc-1 cells and the successful release of DOX when compared to the nanocarrier without modifications. Therefore, this study highlighted the potential of aptamer-modified polymeric micelles being effectively employed for the target delivery of anticancer drugs to treat pancreatic cancer in the near future [169]. Fan et al. also modify polymeric micelles composed only with cellular membrane-disruptive molecules to achieve a long circulation and pH-sensitive nanoparticles as a strategy for accelerating the infiltration of therapeutics, like gemcitabine, through the stromal barrier. In a three-dimensional spheroid with a shell of fibroblast NIH-3T3 cells cultured over a core of pancreatic BxPC-3 cells, these nanoparticles showed acid-activated cytotoxicity to equally cancerous and fibroblast cells through the stromal barrier, being this activity based on the disruption caused to cell membrane integrity by the acidic medium. So, this acid-activatable nanoparticle showed inhibition of tumor growth, suppression of the expression of extracellular matrix components, and activated cancer-associated fibroblasts without causing significative adverse effects in non-target cells, acting as a potential strategy for improving the efficacy of pancreatic cancer treatment [127].

Polymeric micelles can also be used as delivery systems for siRNA-based therapies for pancreatic cancer. In 2019, Xin et al. developed a micelle using the synthetic polymer poly(ethylene glycol)–poly[aspartamidoethyl(p-boronobenzyl) diethyl ammonium bromide] (PEG-B-PAEBEA) for co-delivery of miR-43a mimic, a small molecule polo-like kinase 1 (PLK1) inhibitor and volasertib (BI6727). In cancer patients with pancreatic cancer, a significant downregulation of the tumor suppressor miRNA-34a and elevated levels of expression of PLK1 were observed, which is associated with the low survival rates of patients. The polymeric micelles obtained an average size of 100 nm and a 10% drug loading capacity of volasertib. They also formed a complex with miR-34a at the N/P ratio of 18 and higher. By injecting pancreatic tumor bearer mice with polymeric micelles encapsulating 5 mg/Kg of volasertib and 1 mg/Kg of miR-34a, it was verified a reduction in tumor volume and a histological examination of major organs suggested negligible systemic toxicity, highlighting the potential of these polymeric micelles to deliver volasertib and miR-34a mimic efficiently, and allowing the use of these components as future chemotherapeutic agents for pancreatic cancer treatment [128].

Dendrimers are highly branched macromolecules, and the functional groups present on their surface can be modified to increase biocompatibility and decrease toxicity. It is possible to complex them with siRNA through electrostatic interactions, such as polycyclic dendrimers (polyamidoamine (PAMAMs) or polypropyleneimine (PPI)), which have a high density of positive charges to their surface. For PAMAMs, which have primary amine groups on their surface and tertiary amine groups within them, they can complex with siRNAs. Thus, the complex formed promotes the cellular uptake of siRNA, and the tertiary amine groups initiate the proton sponge effect to increase the endosomal release of siRNA [170]. The in vitro characterization and anti-cancer effect of siRNA delivered by PAMAM dendrimers against human telomerase reverse transcriptase (hTERT) was also evaluated in mouth cancer. Liu et al. concluded that siRNA-hTERT dendriplexes, applied by intratumoral administration, inhibited cell growth and apoptosis in vitro, as well as inhibited tumor growth in vivo in the xenograft model. It also found that there was a decrease in the expression of hTERT proteins in tumors [171].

Wang et al. also studied the possibility of using dendrimers to circumvent the dense fibrotic stroma in the PDA, which is difficult for drug diffusion into the tumor. A dendrimer encapsulating camptothecin, a topoisomerase inhibitor, was developed to penetrate the PDA tumors by γ-glutamyl transpeptidase (GGT)-triggered cell endocytosis and transcytosis. The dendrimer-drug complex was formulated by covalent attachment of camptothecin to the PAMAM dendrimers through a ROS-sensitive linker followed by surface modification with glutathione. The dendrimer-drug complex showed high antitumor activity in multiple mice tumor models, compared to the most standard chemotherapeutic drug used for pancreatic cancer treatment, GEM, demonstrating new potential treatments with new drugs and delivery systems for pancreatic cancer [129].

Tong et al. developed a polymeric system that accommodated the hydrophobic drug GEM in its cavity. This is a pH-sensitive polymer synthesized by conjugating N, N-dipentylethyl moieties, and monomethoxylpoly(ethylene glycol) onto PAMAM dendrimer. These nanoparticles exhibited the ability to facilitate the deep delivery of GEM into the tumor parenchyma as they present as nanoparticles (designated as SPN@Pro-Gem) with a size of about 120 nm at neutral pH, and which, in the pH of the tumor environment, transform into small particles (≈8 nm) [130].

Other synthetic polymers used are Poly Lactic-co-Glycolic Acid (PLGA) and PEG [172,173]. In 2020, Akhter et al. encapsulated naringenin (NARG) in PLGA nanoparticles to enhance the cytotoxic effect of pancreatic cancer. The resulting nanoparticles presented an average particle size of 12.45 nm, a PDI value of 0.132 ± 0.026, and a zeta potential of −20.5 ± 2.5 mV. An in vitro release study showed an initial burst followed by a sustained released profile reaching a cumulative release percentage of 85.67 ± 6.23%. The cytotoxicity of this system against pancreatic cancer cell lines was also evaluated, showing that the NARG nanoparticles presented a higher cytotoxic value when compared to the drug-free form [174]. Jamil et al. produced PLGA nanoparticles loading simultaneously GEM and simvastatin for the treatment of pancreatic cancer. Nanoparticles were formulated by double emulsion technique through a solvent evaporation method and optimized. In in vitro studies, the cell toxicity evaluation of these encapsulated drugs revealed a lower IC50 in comparison to the free drug. Moreover, the bioactivity of GEM and simvastatin increases by 1.4-fold and 1.3-fold, respectively, when loaded in PLGA nanoparticles in comparison to the free drug [175]. Mousa et al. analyzed the anticancer efficacy of the natural products 3, 3′-diindolylmethane (DIM), and ellagic acid (EA) encapsulated into PLGA-PEG nanoparticles. These compounds have already demonstrated anticancer efficacy against several cancer types. However, DIM is insoluble. In the end, both compounds revealed a rapid suppression of pancreatic cancer cell proliferation in 24 h (*p* < 0.01), while the non-encapsulated DIM and EA did not show any significant effect on cancer cell viability or cell proliferation. Using a tumor implant model of pancreatic cancer cells, the results revealed a superior suppression of tumor weight (*p* < 0.01), tumor cell viability, and tumor angiogenesis (*p* < 0.01) for DIM and EA encapsulated and their combinations versus DIM or EA alone [131]. Later, Jung et al. describe a siRNA-loaded PLGA nanoparticle targeting programmed death-ligand 1 (PD-L1) for tumor immunity activation and pancreatic cancer growth reduction. In a humanized preclinical model, the system considerably leads to more apoptotic tumor cells and suppressed pancreatic tumor growth. Multiplex immunofluorescence analysis showed comparable immune cell compositions in control and nanoparticles-treated tumors but a higher Granzyme B expression in nanoparticles-treated tumors, suggesting higher activity of NK or cytotoxic T cells. So, this strategy is revealed to be a potential immunotherapeutic agent for pancreatic cancer [132].

Together, these results showed that polymer nanoparticles may improve the drug’s physical and chemical properties as well as their delivery to the cancer cells concerning the drugs alone.

### 7.4. Inorganic Nanoparticles

Inorganic nanoparticles have come to aid in the diagnosis and treatment of various pathologies. Inorganic nanocarrier systems have contributed to the delivery of drugs, while the inorganic nanoparticles controlled by radiofrequency came to provide a novel approach to the treatment of cancer [176]. The only limitations of this type of nanoparticle are biocompatibility and immunogenicity, but both can be modified by suitable coatings of particles. Iron oxide nanoparticles represent significant advantages such as price since they are cheap to produce, have physical and chemical stability biocompatibility, and are environmentally safe [177].

The most generally utilized iron oxide magnetic nanoparticles have an iron oxide core surrounded by a biocompatible surface coating that demonstrates stability in the physiological environment. The functionalization of this nanoparticle surface by binding functional groups of ligands allows the drug to be directed to a specific target cell. Several studies have already been carried out in which the surface of nanoparticles of iron oxide was modified using cytotoxic drugs, such as DOX, catechin-dextran, and paclitaxel [97]. In a previous study, catechin-dextran nanoparticles were conjugated to Endorem^®^ (FDA-approved drug), showing a rise in the intracellular concentration of the drug compared to the free drug. This formulation was evaluated in MIA PaCa-2 under a magnetic field and caused 98% apoptosis. These results suggested that this conjugation enhanced the antitumor activity of Endorem^®^ and provided a novel delivery system specific for cell tumors driven by magnetic fields [97].

Khan et al. formulated a superparamagnetic iron oxide nanoparticle (SPION) encapsulating curcumin, a nontoxic, anti-cancer compound, for a synergistic effect with GEM to treat pancreatic cancer. It demonstrated an effective delivery of curcumin by the nanocarriers into the tumor cells and, at the same time, a rise in the uptake of GEM and its efficacy. Co-treatment of encapsulated curcumin in SPION and standard GEM treatment decreased tumorsphere formation. In vivo studies in model pancreatic cancer mice showed that elevated levels of curcumin were found in the pancreas, whereas GEM reduced tumor growth and metastasis. These results suggest that curcumin encapsulated in SPION has enormous potential for future therapeutic use in the management of pancreatic cancer [133].

Albukhaty et al. also developed SPION, but this time, they were coated with dextran and conjugated with folic acid to improve specific target delivery and uptake of vinblastine (VBL) in PANC-1 pancreatic cancer cells. The nanoparticles obtained were spherical, without apparent aggregation, and had a mean size of 74 ± 13 nm, a zeta potential of −45 mV, and a polydispersity index of 0.080. This novel carrier system also exhibited low cytotoxicity against healthy cells and high apoptosis values for PANC-1 pancreatic cancer cell lines, preventing those cell proliferation [134].

In 2016, Liu et al., using animal models of pancreatic cancer, enhanced irinotecan loading using mesoporous silica nanoparticles (MSNs), which revealed an improved drug efficacy by increasing drug concentrations at the tumor site compared to the conventional liposomal formulation. Additionally, this system showed a reduction in irinotecan toxicity by reducing systemic pressure leakage of the drug. Given that irinotecan has a high contribution to the toxicity of the four-drug regimen of FOLFIRINOX, these authors considered this system to have the potential to be applied in the FOLFIRINOX program [68]. Tarannum et al. also used MSNs as a platform for the target-specific, spatiotemporal, ratiometric, and safe co-delivery of GEM and cisplatin. By a systemic administration of these nanoparticles in a genetically engineered PDA mouse model, the group found synergistic therapeutic outcomes based on the nanoparticles’ redox-responsive controlled delivery and in situ differential release of GEM/cisplatin drugs to overcome the resistance to platin-based drugs. Thus, this platform might suppress tumor growth and remove the off-target toxicities of a highly toxic chemotherapy combination [135].

Patra et al. developed a gold nanoparticle delivery system containing the targeting agent cetuximab and the anticancer drug gemcitabine. Applying this formulation of nanoparticles both through in vitro assays and in orthotopic pancreatic tumor growth in vivo assay, this group obtained a significant decrease in pancreatic tumor cell proliferation [68].

A clinical trial using colloidal gold nanoparticles with surface-bound recombinant tumor necrosis factor and PEG (CYT-6091) in patients with an advanced solid pancreatic tumor has been shown to selectively target tumor tissue. There were, however, associated adverse effects, such as lymphopenia, hypoalbuminemia, electrolyte disturbances, and disorders in liver enzymes, still not very marked and did not specify any overall survival. These results suggested that the CYT-6091 formulation selectively targets pancreatic cancer, which may be beneficial in delivering chemotherapeutic agents, but further studies are needed to specifically understand the effect in terms of survival rates [72].

In another study, the gold nanoparticles were shown to be biologically viable and highly adaptable for conjugation to any compound having a high functionality. Conjugating gold nanoparticles with GEM demonstrated that there was significant inhibition of pancreatic cancer cell growth in vivo and in vitro with virtually no toxicity [10].

**Figure 7:**
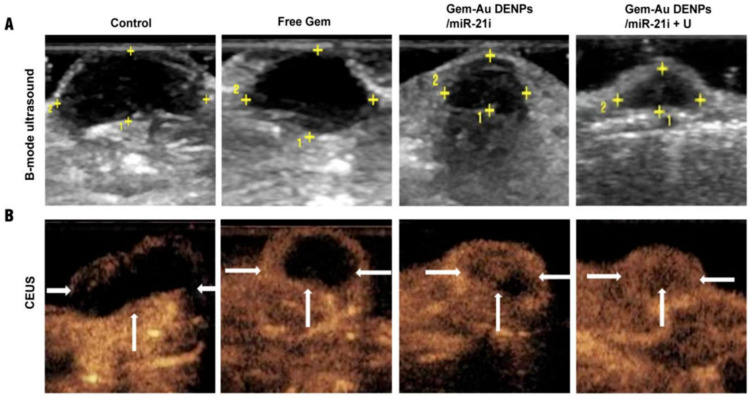
(**A**) shows a B-mode ultrasound image, with the yellow plus signs indicating the tumor region. (**B**) shows a contrast-enhanced ultrasound (CEUS) image is presented. The CEUS imaging reveals abundant intra-tumoral blood flow and a smaller tumor volume in the Gem-Au DENPs/miR-21i + U group. Adapted with permission from [136].

Lin et al. also studied the potential of co-delivery of GEM and miR-21 inhibitors with dendrimer-entrapped gold nanoparticles (AU-DNPs) to treat pancreatic cancer. It was observed that co-delivering GEM and miR-21, using AU-DNPs as nanocarriers, showed a decrease of IC_50_ values of 13-fold when compared to the delivery of these components in their free form. In further in vivo treatments, significant tumor volume reduction and a rise in blood perfusion of xenografted pancreatic tumors (Figure 7) [136].

In another study, Coelho et al. used pegylated gold nanoparticles (PEGAuNPs) as nanocarriers for a combination of DOX, a highly effective antineoplastic agent against diverse cancer phenotypes, and varlitinib, a potent tyrosine kinase inhibitor with high antitumor activity, to perform an in vitro evaluation of their cytotoxicity against pancreatic tumor and healthy cell lines. It was concluded that by encapsulating these two drugs into PEGAuNPs, the toxicity of these drugs against healthy pancreatic cell lines was reduced, while the toxicity for cancer line S2-013s increased, decreasing the survival rate of the pancreatic cancer cell lines. This study shows the promising potential of gold nanoparticles to improve specific targeting cancer therapy and overcome limitations regarding low bioavailability [137].

Hafiz et al. present a nanoplatform as a drug delivery strategy for photosensitizers based on a new liquid metal mixture that controls the tumor microenvironment to reach photodynamic therapeutic effects in pancreatic cancer. The liquid mixture based on eutectic gallium-indium nanoparticles was conjugated with hyaluronic acid, water-soluble cancer targeting ligand, and benzoporphyrin derivative, a photosensitizer, via a simple green sonication method, resulting in sphere-shaped nanoparticles with core–shell structure, high biocompatibility, and stability. These nanoparticles showed improved cellular uptake and targeting competence and resulted in significantly higher intracellular ROS. Moreover, near-infrared light activation of nanoparticles revealed their potential to effectively destroy cancer cells once their single oxygen generation capability. Lastly, in vivo studies, eutectic gallium-indium nanoparticles conjugated with hyaluronic acid and benzoporphyrin derivative caused tumor regression and resulted in 2.3-fold higher necrosis than the control, revealing this nanoplatform as a good vehicle for photodynamic therapy and a new strategy for enhanced cancer therapy [178].

Overall, inorganic nanoparticles, such as iron oxide and gold nanoparticles, have shown promise in pancreatic cancer diagnosis and treatment. They offer advantages like biocompatibility, stability, and tunable surfaces for drug delivery. Modified iron oxide nanoparticles have been used for targeted drug delivery in pancreatic cancer, enhancing therapeutic efficacy. Gold nanoparticles have been explored for the co-delivery of chemotherapy drugs and miRNA inhibitors, resulting in reduced toxicity to healthy cells and improved anticancer effects. Additionally, novel liquid metal-based nanoplatforms have demonstrated efficient photodynamic therapy for pancreatic cancer, showing potential for enhanced cancer treatment. These studies highlight the potential of inorganic nanoparticles in developing effective and targeted therapies for pancreatic cancer.

## 8. Toxicity Concerns in Nanoparticle Delivery Systems

The toxicity associated with this type of delivery system may occur because of the size, composition, or load of the nanoparticles. In general, the particles of smaller dimensions present greater toxicity compared to the larger ones with the same chemical and crystalline structure and composition. Because they are so small, they easily cross biological barriers and reach their target organs. However, smaller nanoparticles can also cross the blood-brain barrier, damage healthy cell membranes, and trigger severe immune responses. In this sense, it is important to achieve a balance between therapeutic effect and toxicity [179,180].

The composition and charge of the nanoparticles also influence their accumulation and toxicity, and the surface charge given by the zeta potential must be addressed. A zeta potential value above (±) 30 mV is desirable because it avoids undesirable aggregation of the particles that, in turn, can result in changes in their physicochemical properties such as distribution, surface-to-volume ratio, and surface activity. These changes may also give rise to their clearance by macrophages or vascular/lymphatic blockage, toxicity, and inflammation. Furthermore, since one of the factors that promote this aggregation is the large surface area, particles with a low surface-area-to-volume ratio are desirable to reduce their toxicity [179].

The main concern related to nanoparticles is the fact that some compositions, such as metal-based, can be toxic. Sometimes, encapsulating material causes increased production of ROS, which can lead to mutations in the DNA. There are also reports that exposure to nanoparticles is associated with asthma, bronchitis, Alzheimer’s disease, and Parkinson’s disease. Several events related to vascularization, such as blood clots, are associated with nanoparticles administered parenterally. Cationic liposomal nanoparticles can interact with the extracellular matrix, serum proteins, and lipoproteins, resulting in aggregation and /or oxidative stress that can cause non-target tissue damage [181].

Although the metallic nanoparticles have a low surface charge at physiological pH, they are prone to aggregate in solution due to their large surface area in relation to size and an intrinsic metallic character. As previously described, in some cases, aggregation is a concern because it can decrease long-term stability [103]. The accumulation of dose-dependent silver nanoparticles was observed in the brain and other organs, suggesting a systemic distribution after oral administration. There was also a significant increase in alkaline phosphatase and cholesterol, indicating hepatotoxicity [182]. Gold nanoparticles can cross the placenta and cause damage to fetal development but are also involved in the induction of reactive oxygen formation and initiation of autoimmunity [183,184]. These gold nanoparticles are more toxic when they have smaller dimensions (1–2 nm), revealing their toxicity in both human cancer cells and healthy cells. Larger gold nanoparticles (4.8–12 nm), on the other hand, show relevant toxicity to cancer cells but little toxicity to healthy cells. Finally, gold nanoparticles larger than 15 nm are considered to be non-toxic [185,186]. Additionally, at the cellular level, toxicity from these nanoparticles is because of the drug they carry, namely through modulation of gene expression, promotion of ROS production at the mitochondrial level, activation of autophagy, or modification of membrane potential [187].

In 2016, liposomal irinotecan was approved for the treatment of advanced pancreatic cancer. However, this treatment did not show the expected results and came with dangerous toxicity, so its use was questioned. So, to reduce the toxicity of this carrier and also increase its therapeutic benefit, a study realized by Liu et al. used silica nanoparticles to encapsulate irinotecan, showing improved accumulation of the compound, superior therapeutic efficacy, and reduced systemic output compared to the liposomal carrier [188].

Gemcitabine combined with erlotinib is usually used to treat pancreatic cancer, but it is associated with significant toxicity. Cai et al. designed a gemcitabine-loaded PLGA nanoparticle with a macrophage membrane coating to reduce drug toxicity and increase the accumulation in the tumor site, even in combination with erlotinib. This platform showed inhibited pancreatic cancer cell proliferation in vitro and in vivo studies [189].

So, since there are several materials applied in the production of nanoparticles, there is an infinite number of combinations of interactions with a high potential for harmful interactions that must be studied and considered to ensure the life and well-being of patients.

## 9. Walkthrough on Pipeline Products

In January 2012, there were already more than 33 approved nano-drugs marketed worldwide [190,191]. In the liposomes field, in addition to DOXil^®^, other nano-drugs have been approved. In 2015, the FDA approved a nanoliposomal formulation of irinotecan called Onivyde^®^ [192]. This drug inhibits topoisomerase I enzyme and is used, combined with 5-fluorouracil and leucovorin, in the treatment of metastatic pancreatic cancer after therapies with GEM [81,182]. A randomized trial was conducted on a series of 417 patients affected by metastatic PDACs. They received the combination of Onivyde^®^ and 5-fluorouracil (5-FU)/Leucovorin with an average of 2 months longer survival and a mean delay in time to tumor growth of 3.1 months when compared to those who received only 5-FU/Leucovorin [68].

No less important, metal-based nanoformulations, such as NanoTherm^®^, approved since 2013, are indicated for glioblastoma, prostate, and pancreatic cancer (intratumoral). It consists of a formulation consisting of nanoparticles of superparamagnetic iron oxide coated with aminosilane. It acts by thermal ablation, injecting the nanoparticles exposed to the magnetic field, causing them to oscillate, thus generating a heatwave directly inside the tumor tissue [193].

Rexin-G^®^, described as the “first targeted molecular genetic injectable medicine”, was approved, blocking the endogenous cyclin G1 protein, disrupting the cell cycle. This drug shows the advantage that it can be used for all metastatic solid tumors, unlike others that cannot be used if they are metastatic [194,195]. Chawla et al. showed that growing doses of Rexin-G in metastatic gemcitabine refractory pancreatic adenocarcinoma were able to increase disease-free survival by a few months, revealing that this targetable injectable retroviral vector is safe and exhibits antitumor activity [196].

Other nanoformulations are still in development. One example is PanDOX, a non-randomized new clinical trial that has been running since June 2021, in phase I perspective now [197]. This system pretends to improve the DOX therapy using Thermosensitive Liposomal DOX (ThermoDOX^®^) and focused ultrasound in the treatment of non-resectable primary pancreatic tumors concerning systemic delivery of free DOX [198,199]. DOX is bound to liposomes, like DOXil^®^, but is formulated with thermal sensible lipids that degrade the bilayer when exposed to high temperatures [81]. Several other clinical trials are underway for combination therapies based on GEM, which blocks cell cycle progression at the G1 / S phase boundary for the treatment of pancreatic cancer. The combination of GEM and Abraxane^®^ for stage II pancreatic cancer is in phase II, and GEM with erlotinib for advanced localized cancer is in phase I, among others [200].

## 10. Conclusions

Pancreatic cancer is one of the oncological pathologies with the highest associated mortality rate, being the main reason for the stage IV diagnosis, where metastasis already exists. Some factors can be used for prognoses, such as tumor size, lymph node involvement, and resection margin status. The treatment of this type of cancer is still a continuous challenge since chemoresistance is very associated with this type of cancer disease. The therapies so far are mostly based on nonspecific chemotherapy agents. However, over the last few years, there has been a progression that has led to increased interest in the development of molecularly designed, targeted therapies for the treatment of pancreatic cancer. Thus, nanotechnology is considered a good strategy to deliver drugs that would allow tumor-directed administration of cytotoxic drugs.

Clinical trials were performed involving albumin, colloidal gold, iron oxide, micelles, and liposomes, among others, and demonstrated that nanoparticles can be used in conjunction with chemotherapeutic agents and others as they increase their efficacy and reduce their toxicity, very present in non-specific chemotherapy. Despite the efficacy demonstrated in vivo and in vitro studies in animal models, more research is needed to understand the long-term side effects of nanoparticle use, particularly in terms of systemic toxicity and clinical translation.

In the future, should we manage to encapsulate a drug with cytotoxic activity specifically tailored for pancreatic tumor cells within non-toxic nanoparticles that can evade immunogenic reactions and target only the desired cells with high specificity, we might overcome the existing challenges in treatment. This advancement could reduce cancer recurrence, enhance the overall survival rate of pancreatic cancer patients, and improve their quality of life. Efforts to overcome physiological barriers in pancreatic cancer should also be made, including early detection, enhanced drug circulation, better tumor penetration, and controlled drug delivery. Overall, this manuscript discusses the main scientific advances in pancreatic cancer treatment by nano-based drug delivery systems, discloses some challenges, and points out some drawbacks that should be valuable in future research in the field.

## Figures and Tables

**Table 1:** International classification of malignant tumors for pancreatic cancer. Adapted with permission from [41].

Primary Tumor (T)		Regional Lymph Nodes (N)		Distant Metastasis (M)	
TX	Primary tumor cannot be assessed	NX	Regional lymph nodes cannot be assessed	M0	No distant metastasis
T0	No evidence of primary tumor	N0	No regional lymph node metastasis	M1	Distant metastasis
Tis	Carcinoma in situ	N1	Regional lymph node metastasis		
T1	Tumor limited to the pancreas, 2 cm or less in greatest dimension				
T2	Tumor limited to the pancreas, more than 2 cm in greatest dimension				
T3	Tumor extends beyond the pancreas but without the involvement of the celiac axis or the superior mesenteric artery				
T4	Tumor involves the celiac axis or the superior mesenteric artery; unresectable primary tumor				

**Table 2:** International classification of malignant tumors: stages of pancreatic cancer. Adapted with permission from [41].

Stage	Primary Tumor	Regional Lymph Nodes	Distant Metastasis
**0**	Carcinoma in situ	No regional lymph node metastasis	No distant metastasis
**IA**	Tumor limited to the pancreas, 2 cm or less in greatest dimension	No regional lymph node metastasis	No distant metastasis
**IB**	Tumor limited to the pancreas, more than 2 cm in greatest dimension	No regional lymph node metastasis	No distant metastasis
**IIA**	Tumor extends beyond the pancreas but without the involvement of the celiac axis or the superior mesenteric artery	No regional lymph node metastasis	No distant metastasis
**IIB**	Tumor limited to the pancreas; Tumor extends beyond the pancreas but without the involvement of the celiac axis or the superior mesenteric artery	Regional lymph node metastasis	No distant metastasis
**III**	Tumor involves the celiac axis or the superior mesenteric artery; unresectable primary tumor	No regional or regional lymph node metastasis	No distant metastasis
**IV**	Tumor limited to the pancreas; Tumor extends beyond the pancreas but without involvement of the celiac axis or the superior mesenteric artery; Tumor involves the celiac axis or the superior mesenteric artery; unresectable primary tumor	No regional or regional lymph node metastasis	Distant metastasis

**Table 3:** Overview of several examples of nanocarriers applied in pancreatic cancer treatment.

Nanocarrier	Load	Characterization	Targeting Moiety	Targeting Cell/Tissue	In Vitro/Vivo Model	Application	Ref.
Liposomes	siRNA against HER-2	Particle size = ~100 nm	TfR antibody receptor	Human pancreatic cancer cell line PANC-1	Murine xenograft model	Pancreatic cancer	[113]
Nanoliposomal system (MM-398)	Irinotecan	Particle size = ~111 nm; PdI = 0.04	Topoisomerase I	Metastatic pancreatic ductal adenocarcinoma (PDA)	Global, phase 3, randomized, open-label trial	2nd line therapy in metastatic PDA	[114,115,116]
Liposome FF-10832	Gemcitabine	Particle size = 79 ± 2 nm	DNA synthesis and ribonucleotide reductase	Pancreatic cancer cell lines	Mice with Capan-1, SUIT-2, and BxPC-3 tumors	Pancreatic cancer	[117]
Liposomes	Collagenase	-	Collagen	Extracellular collagen stroma matrix	Mice-bearing PDA tumors	Disassemble the collagen stroma matrix	[96]
Liposomes (BS-LipoIRI)	Irinotecan	Particle size = 125 nm; drug encapsulation efficiency = 80.95%	Epidermal growth factor receptor (EGFR) and fibroblast activation protein bispecific antibody	Pancreatic tumor cells and tumor-associated fibroblasts	Eight-week-old SCID mice	Human Pancreatic Tumor	[118]
Solid lipid nanoparticles (SLN)	Gemcitabine	Particle size = 603 ± 19 nm; entrapment efficiency = 68.3 ± 4.8%	DNA synthesis and ribonucleotide reductase	Patient-derived primary pancreatic cancer cell lines	MiaPaCa-2 and PPCL-46 cell lines	Human Pancreatic Tumor	[119]
SLN	Aspirin and Curcumin	Particle size of 150 and 250 nm; encapsulation efficiency of 85 and 69%	Cyclooxygenase-2; anti-inflammatory/anti-cancer effect	Pancreatic cancer cells	MIA Paca-2 and Panc-1 cell lines	Chemoprevention of pancreatic cancer	[120]
Cationic Nanostructured lipid carriers (NLC)	microRNA miR-34a and miR-143/145	Nanovector size ~100 nm	SIRT1, CD44, aldehyde dehydrogenase, KRAS2 and Ras-responsive element binding protein-1 (RREB1)	Pancreatic cancer xenograft model	MiaPaCa-2 subcutaneous xenografts	Pancreatic cancer	[121]
Hyaluronic acid-coated NLCs	Gemcitabine and Baicalein (BCL)	-	DNA synthesis and ribonucleotide reductase	Human pancreatic adenocarcinoma cell lines	AsPC1 cells lines	Human pancreatic adenocarcinoma	[122]
Lipid-polymer hybrid nanoparticle	Gemcitabine and HIF1a siRNA	-	DNA synthesis and ribonucleotide reductase	-	Subcutaneous and orthotopic tumor models	Pancreatic cancer	[123]
Albumin nanoparticles encapsulated in modified thermosensitive liposomes	Paclitaxel	Particle size = 123.9 ± 1.9 nm	Mitotic arrest in the cell cycle at the mitotic phase	Tumor mouse models	Pan 02 subcutaneous and orthotopic tumor	PDA	[124]
Gelatin nanoparticles marked with a redox-responsive EGFR	Gemcitabine	-	DNA synthesis and ribonucleotide reductase	Orthotopic pancreatic cancer model	Panc-1 human pancreatic ductal adenocarcinoma cells	PDA	[125]
Nanobioconjugate chitosan-based	Gemcitabine and anti-EGFR antibodies	Encapsulation rate = 91.63%	DNA synthesis, ribonucleotide reductase and EGFR	Human pancreatic cancer cells	Human pancreatic cancer cell lines SW1990	Pancreatic cancer	[16]
Chitosan nanoparticles	Quercetin and 5-fluorouracil	Particle size = 402 ± 52 nm; entrapment efficiency = 95 and 75%	Chromosome segregation and organization	Primary pancreatic cancer cell line and mouse cell line	MiaPaCa2 and primary mouse fibroblast cell line	Pancreatic cancer	[126]
Polymeric micelles with cellular membrane-disruptive molecules	Gemcitabine	Particle size from 107 ± 11.9 to 163.1 ± 13.1 nm	DNA synthesis and ribonucleotide reductase	Human pancreatic cancer cells	3D spheroid, shell of fibroblast; NIH-3T3 cells over pancreatic BxPC-3 cells	Pancreatic cancer	[127]
Polymeric micelles	microRNA miR-34a and volasertib (BI6727)	Particle size = 100 nm; drug loading capacity = 10%	Suppression of Bcl-2	Pancreatic cell lines	Orthotopic pancreatic tumor-bearing NSG mice, MIA PaCa-2R cell line	PDA	[128]
PAMAM dendrimers	Camptothecin	Particle size = ~20 nm	Topoisomerase inhibition	Mice tumor models	Patient-derived PDA xenograft and orthotopic PDA cell xenograft	PDA	[129]
Polymeric system onto PAMAM dendrimer	Gemcitabine	Particle size = ~120 nm	DNA synthesis and ribonucleotide reductase	Pancreatic cell lines and mice bearing Panc02 pancreatic tumor xenografts	Adherent Panc02 cells, 3D multicellular spheroids (MCSs) and ICR mice	PDA	[130]
Poly Lactic-co-Glycolic Acid (PLGA) nanoparticles	Naringenin (NARG)	Particle size = 150.45 ± 12.45 nm; PDI = 0.132 ± 0.026; Zeta potential = −20.5 ± 2.5 mV	Free radical scavenging activity	Pancreatic cell lines	-	Pancreatic adenocarcinoma	[124]
PLGA nanoparticles	Gemcitabine and simvastatin	Particle size = 258 ± 2.4 nm; PDI = 0.32 ± 0.052; zeta potential = −12.5 mV	DNA synthesis and ribonucleotide reductase; avoidance of translation of pancreatic intraepithelial neoplasia to PDA	Pancreatic cell lines	MCF-7 and MIA PaCa-2 cells; Wistar rats	PDA	
PLGA-PEG nanoparticles	3, 3′-diindolylmethane (DIM), and ellagic acid (EA)	Particle size = 180–210 nm	Apoptosis induction	Human pancreatic cancer cell line	SUIT2 expressing firefly luciferase (SUIT2-Luc)	Pancreatic cancer	[131]
PLGA nanoparticle	siRNA	Particle size = 188.5 ± 1.2 nm	Programmed death-ligand 1 (PD-L1	PDA tumor-bearing humanized mice	-	Pancreatic cancer	[132]
Superparamagnetic iron oxide nanoparticle (SPION)	Curcumin	Particle size = 120 to 140 nm; zeta potential = −17 to −20 mV	Anti-inflammatory/anti-cancer effect	Model pancreatic cancer mice	-	Pancreatic cancer	[133]
SPION coated with dextran and conjugated with folic acid	Vinblastin	Particle size = 74 ± 13 nm; zeta potential = −45 mV; polydispersity index = 0.080	Mitotic arrest in the cell cycle at the mitotic phase	Pancreatic cell lines	PANC-1 pancreatic cancer cells	Pancreatic cancer	[134]
Mesoporous silica nanoparticles (MSNs)	Gemcitabine and cisplatin	Particle size between 120 to 1890 nm	DNA synthesis and reparation	PDA cell lines	PDA.MUC1 Mouse Model	PDA	[135]
Dendrimer-entrapped gold nanoparticles (AU-DNPs)	Gemcitabine and miR-21 inhibitors	Particle size between 154 to 276 nm	DNA synthesis	Xenografted pancreatic mouse tumors	SW1990 cells	Pancreatic cancer	[136]
Pegylated gold nanoparticles (PEGAuNPs)	Doxorubicin and varlitinib	Particle size = 24 ± 1 nm; zeta potential = −41 ± 2 mV	Topoisomerase-II-mediated DNA repair; tyrosine kinase	Pancreatic tumor	MIA PaCa-2, S2-013 cells, and hTERT immortalized human cell	Pancreatic cancer	[137]

## Abbreviations and Acronyms

ANS—Autonomic Nerves System; anti-EGFR—Anti-epidermal growth factor receptor; ApoB—Apoprotein B; Au-DNPs—Dendrimer-entrapped gold nanoparticles; BCL—Baicalein; BS-LipoIRI—Anti-fibroblast activation protein bispecific antibody-targeted liposomal irinotecan; CCK—Cholecystokinin; CEA—Carcinoembryonic antigen; CgA—Chromogranin A; DIM—3′-Diindolylmethane; DOX—Doxorubicin; EA—Ellagic acid; EGFR—Epidermal growth factor receptor; GEM—Gemcitabine; GEM-SA—GEM-stearic acid lipid prodrug; HA—Hyaluronic acid; HA-AA-BCL—Hyaluronic acid-amino acid-baicalein; hTERT—Human telomerase reverse transcriptase; KRAS—Kirsten rat sarcoma virus; MCNs—Mucosal cystic neoplasms; miRNAs—Interference-based RNA; MSNs—Mesoporous silica nanoparticles; NARG—Naringenin; NETs—Neuroendocrine tumors; PAMAMs—Polyamidoamine; PanIN—Pancreatic intraepithelial neoplasm; Pasc-1 cells—Human pancreatic tumor cells; PCL—Polycaprolactone; PDA—Pancreatic ductal adenocarcinoma; PD-L1—Programmed death-ligand 1; PEG—Poly (ethylene glycol); PEGAuNPs—Pegylated gold nanoparticles; PET—Positron emission tomography; PLA—Poly (lactic acid); PLGA—Poly Lactic-co-Glycolic Acid; PLK1—Polo-like kinase; PPI—Polypropyleneimine; ROS—Reactive oxygen species; Si-HIF1a—HIF1a siRNA; SLN—Solid lipid nanoparticles; TfR—Transferrin receptor; UMMD—Ultrasound-mediated microbubble destruction.
